# An integrative systematic review of employee silence and voice in healthcare: what are we really measuring?

**DOI:** 10.3389/fpsyt.2023.1111579

**Published:** 2023-05-25

**Authors:** Olga Lainidi, Mimmi Kheddache Jendeby, Anthony Montgomery, Christos Mouratidis, Konstantina Paitaridou, Clare Cook, Judith Johnson, Eirini Karakasidou

**Affiliations:** ^1^School of Psychology, University of Leeds, Leeds, United Kingdom; ^2^University of Gothenburg, Gothenburg, Sweden; ^3^Department of Psychology, Northumbria University, Newcastle, United Kingdom; ^4^School of Psychology, Mediterranean College, Thessaloniki, Greece; ^5^Department of Psychology, Panteion University, Athens, Greece

**Keywords:** employee silence, employee voice, healthcare, patient safety, speaking-up, withholding information

## Abstract

The history of inquiries into the failings of medical care have highlighted the critical role of communication and information sharing, meaning that speaking up and employee silence have been extensively researched. However, the accumulated evidence concerning speaking-up interventions in healthcare indicates that they achieve disappointing outcomes because of a professional and organizational culture which is not supportive. Therefore, there is a gap with regard to our understanding of employee voice and silence in healthcare, and the relationship between withholding information and healthcare outcomes (e.g., patient safety, quality of care, worker wellbeing) is complex and differentiated. The following integrative review is aimed at addressing the following questions; (1) How is voice and silence conceptualized and measured in healthcare?; and (2) What is the theoretical background to employee voice and silence?. An integrative systematic literature review of quantitative studies measuring either employee voice or employee silence among healthcare staff published in peer-reviewed journals during 2016–2022 was conducted on the following databases: PubMed, PsycINFO, Scopus, Embase, Cochrane Library, Web of Science, CINAHL and Google Scholar. A narrative synthesis was performed. A review protocol was registered on the PROSPERO register (CRD42022367138). Of the 209 initially identified studies for full-text screening, 76 studies met the inclusion criteria and were selected for the final review (*N* = 122,009, 69.3% female). The results of the review indicated the following: (1) concepts and measures are heterogenous, (2) there is no unifying theoretical background, and (3) there is a need for further research regarding the distinction between what drives safety voice versus general employee voice, and how both voice and silence can operate in parallel in healthcare. Limitations discussed include high reliance on self-reported data from cross-sectional studies as well as the majority of participants being nurses and female staff. Overall, the reviewed research does not provide sufficient evidence on the links between theory, research and implications for practice, thus limiting how research in the field can better inform practical implications for the healthcare sector. Ultimately, the review highlights a clear need to improve assessment approaches for voice and silence in healthcare, although the best approach to do so cannot yet be established.

## 1. Introduction

Healthcare organizations are unique, in the sense that the services provided involve risks that can range from minor nuisances to life-threatening and/or fatal consequences for patients, which puts a lot of pressure on healthcare professionals, staff, administrators, boards and policymakers. The reality of day-to-day practices in healthcare was brought into sharp focus globally during the recent COVID-19 pandemic, which highlighted the fragility of healthcare systems globally revealing the considerable stress experienced by healthcare staff (1). Both past and more recent inquiries into the failings of care have highlighted the critical role of communication and information sharing, indicating that speaking up and voicing concerns is an integral part of safe clinical practice (2–5). The same inquiries, however, have shown that (a) staff’s voiced concerns are frequently not acted upon until a disaster point is reached, (b) professionals with high calling intensity (i.e., professions with psychological contracts that encourage presenteeism even when employees are ill) frequently remain silent on critical issues related to patient safety and/or unprofessional behavior, and (c) whistleblowing is still considered the most “successful” channel to address systemic and organizational problems that have remained unresolved for a long time. Silence in health care has been related to concealing personal errors and covering errors made by others (6, 7), as well as reduced patient safety (8).

Two influential definitions of employee voice are those of Morrison (9) which describes “*employee voice as informal and discretionary communication of ideas*, *suggestions, concerns, problems, or opinions about work-related issues, with the intent to bring about improvement or change*” (p. 80) and that of LePine and Van Dyne (10) with voice being a term for “*speaking out and challenging the status quo with the intent of improving the situation*” (p. 853). More recently, the research on employee voice has been enriched by the increased interest in behaviors of withholding information from colleagues or superiors in the workplace, known as “employee silence”. One of the most influential definitions of employee silence has been provided by Pinder and Harlos (11): *“an employee’s intentional withholding of genuine expression about behavioral, cognitive, and/or affective assessments of organizational conditions to organizational members who seem capable of changing the situation”*. Tangirala and Ramanujam (12) define employee silence as *“employees’ intentional withholding of critical work-related information from other members of their workgroup”* (p. 41). However, it remains unclear what is included in the terms “employee voice” and “employee silence” in healthcare, as they can sometimes be discussed in terms of safety voice and safety silence (i.e., speaking about patient safety/patient advocacy or concealing information related to patient safety, respectively); how employee voice/silence fits within the theoretical literature on organizational culture and behavior in healthcare; and/or whether it extends to the professional culture and identity of healthcare staff. This is also related to the fact that although there is a general agreement that employee silence refers to withholding information and employee voice refers to sharing information in the workplace, any attempt to operationalize employee voice/silence reveals difficulties and challenges in identifying what should be considered voice/silence. This can in turn affect the way voice/silence are measured. For example, voice is defined as a discretionary behavior, in that individuals choose whether to engage in verbally expressing themselves or not at any particular moment with this being affected by a variety of factors (9). Similarly, the definitions of silence presented previously define silence as the “intentional withholding”. In healthcare though, the notion that voice/silence is a discretionary behavior can be easily misinterpreted, especially if we take into account the type of information that is often likely to be conveyed in healthcare: a concern related to patient safety and/or quality of care. Thus, the content and the context of speaking-up can differentiate the extent to which silence or voice are discretionary, as concealing a medical error for instance has ethical, moral and legal ramifications.

Recent literature has suggested that, although on a lexical level employee silence and employee voice seem to be opposite terms, they might also be distinct concepts with different antecedents (13). Moreover, silence and voice can occur at the same time, meaning that employees might be speaking up in some situations (or regarding specific issues), but withholding their voices in other situations. For example, the definition by Pinder and Harlos (11) specifies the withholding of “genuine expression”; this means that even in situations where employees engage in speaking, it cannot be ascertained that they are not engaging in any form of withholding voice (e.g., instead of speaking up about the unprofessional behavior of a colleague they may share a generally neutral comment on workplace behavior). This is particularly relevant to healthcare organizations, where a significant amount of the information shared (or withheld) is frequently related to patient safety and quality of care, which involves the interests not only of the healthcare professionals and the organization itself, but also those of the patients and their families—which has also been discussed as a conflict of interest (14).

The increasing empirical evidence regarding speaking-up in healthcare suggests that silence is the norm while voicing concerns is met with negative consequences for employees (15–18). For example, employee silence has been linked to employee well-being in the literature (19). In terms of understanding how silence/voice links with different outcomes for employees, we build upon the example of employee well-being, and more specifically burnout (19). The Job Demands-Control Model (JD-C) (20, 21), the Job Demands-Resources Model (JD-R) (22, 23) and the Conservation of Resources Model (COR) can help advance our understanding of employee voice behaviors in healthcare organizations (24, 25) and their links to employee outcomes. Both the JD-C and JD-R models view burnout through the lens of a mismatch between demands and resources; in this context employee silence could be evidence of the mismatch while employee voice could be evidence of a better fit. COR emphasizes the tendency of individuals and groups to always aim to obtaining, retaining, fostering, and protecting the resources they centrally value. One of the main principles of the COR theory suggests that when employees’ resources are (almost) depleted, individuals are more likely to enter a defensive mode to preserve the remaining resources or to seek for alternative survival/adaptation strategies if previous experiences were found to be maladaptive and consuming; these defensive modes can be sometimes aggressive and/or irrational. A common response, for example, might be defensive withdrawal, allowing the individuals to gain time to regroup, wait for help and allow the stressor to pass (26). Viewed through this lens, silence could be the result of an employee moving into a defensive mode in response to depleted resources.

Greater understanding of how employee voice/silence among healthcare professionals is conceptualized and measured is proposed as a potentially effective way to identify what is considered employee voice/silence in healthcare. It has been argued that withholding concerns is the norm in healthcare (27) and while healthcare organizations might share some common antecedents of voice behaviors with other industries (e.g., fear of retaliation or losing one’s job; not wanting to risk relationships with colleagues etc.), there are specific characteristics of healthcare education and professional culture that should be taken into account when examining voice among healthcare workers (28). Meta-analytic findings suggest that interventions to improve speaking up in healthcare achieve disappointing results (29). In order to better understand why this is happening, it is necessary to understand how employee silence and voice are operationalized in healthcare and whether there is a need for a new framework adapted for healthcare organizations specifically. By synthesizing and/or critiquing existing research on employee voice/silence among healthcare professionals, an Integrative Systematic Review can offer new insights and new ways of understanding the phenomenon (30). Therefore, the purpose of this integrative review was to explore the following questions;

(1) How are employee voice and employee silence conceptualized and measured in healthcare?

(2) What is the theoretical background to employee voice and silence in healthcare?

## 2. Method

### 2.1. Methodology

The methodology used in the present review involved an integrative systematic literature review. Reviews should meet the same standards of methodological rigor as primary research (31) and given that it is complex to combine various methodologies into one review it becomes even more important to use an explicit and systematic method to avoid inaccuracy and biases (32). Conventional systematic reviews and meta-analyses are the preferred methodology when the available data is appropriate. However, when a phenomenon requires clarification and insight, which involves a more interpretive synthesis of existing literature, an IR which combines the best elements of a systematic review and narrative review is more appropriate. According to Greenhalgh et al. (33) the systematic review format has been erroneously defined as a universal gold standard, partly because the term “narrative review” is frequently misunderstood, misapplied and unfairly dismissed. Toronto and Remington’s (34) six steps method served as a framework for this Integrative Review (IR). The six steps are: (1) problem formulation, (2) data collection *via* systematic literature search, (3) evaluation of data points by analyzing quality and relevance of selected literature, (4) data analysis and interpretation, (5) presentation of results *via* discussion and conclusion and finally, and (6) dissemination of findings. This framework is built on Cooper’s (31) approach for conducting IRs with a transparent and rigorous systematic approach to reviewing the literature.

### 2.2. Problem formulation

There is a gap in our understanding of employee voice and silence in healthcare, and the relationship between withholding information and healthcare outcomes (e.g., patient safety, quality of care, worker wellbeing) is complex and differentiated. While there is empirical evidence suggesting some understanding of certain aspects of voice/silence among working populations in general (e.g., the relationship between employee silence and employee well-being) (19), the uniqueness of the healthcare sector means that a tailored approach is needed to further understand what drives voice/silence in healthcare; how it impacts quality of care; and what factors need to be addressed in order to identify suitable and relevant practical implications. This highlights the needs to review what measures are used to assess employee voice/silence in healthcare as well as whether the existing research is driven by distinct theoretical frameworks. The complexity of investigating the phenomena of employee silence and employee voice in healthcare is suited to the holistic approach of the IR. According to Toronto and Remington (34), the IR approach looks more broadly at a phenomenon of interest than a systematic review and allows for diverse research, which may contain theoretical and methodological literature to address the aim of the review. Moreover, the IR approach supports a wide range of inquiry, such as defining concepts, reviewing theories, and analyzing methodological issues.

### 2.3. Data collection *via* systematic literature search

A review protocol was registered on the PROSPERO register (CRD42022367138). An electronic database search for the period from January 2016 to January 2022 was conducted by two researchers (OL and MKJ), independently. Librarians were also consulted throughout the search process to ensure its quality (34). The following electronic databases were used: PubMed, PsycINFO, Scopus, Embase, Cochrane Library, Web of Science, CINAHL and Google Scholar. Additionally, analysis of references lists of retrieved studies and manual scoping was conducted. Given the large amount of literature on the subject and the need to analyze the most recent publications, the search was limited from January 2016 to January 2022. This condensed time-period was wide enough to provide an appropriate snapshot of the literature and dense enough to examine the range of conceptualizations, measures and potential implications in the literature. Initially the search period was set at 10 years, but the amount of research papers became unwieldy making the review process unmanageable, so a decision was made to analyze the most recent papers in the last 6 years. The choice of 6 years was based on the need to capture a manageable number of papers, as well as the following factors: (a) the amount of research around employee silence/voice in healthcare has peaked since 2016 and even more so after the COVID-19 pandemic; (b) the way that employee silence/voice has been measured in healthcare has been recycled over the last 10–15 years.

Keywords used in the systematic search were identified by reviewing article examples and results found in preliminary searches (34). The keywords identified were then put together into a search string (for the search strings used for the different databases see Supplementary material 1); for example:

(“Employee silenc*” OR “Employee voic*” OR “Organizational silenc*” OR “Organisational silenc*” OR “Organizational voic*” OR “Organisational voic*” OR “Silence behaviour” OR “Voice behaviour” OR “Voice behavior” OR “Speak up” OR Speak-up OR “Prohbitive voic*” OR “Promotive voic*” OR “speak-up related climate” OR “silence culture” OR Concealment OR “Truth disclosure” OR “Transparency” OR “Error concealment” OR Confidentiality OR “medical error*” OR “rais* concerns” OR Whistleblow* OR Whistle-blow* OR “blowing the whistle”)AND (Healthcare OR “Health care” OR “health organization” OR “health organisation” OR “health service” OR “medical service” OR hospital OR hospitals OR “primary care” OR “healthcare employee*” OR “health care employee*” OR “health personnel” OR “health employee” OR nurs* OR physician* OR doctor* OR medic* OR “patient safety”)

The search included both title and abstract. The benefit of the IR approach is that it creates conditions for a comprehensive search of literature (30) and a broad search was conducted. Five inclusion criteria were applied. These were: (1) the paper had to be in the English language, (2) the paper had to be primarily based on quantitative research, (3) only peer reviewed journal papers were included, (4) voice and/or silence had to be measured quantitatively, either directly *via* silence or voice scales or indirectly *via* other scales incorporating voice or silence constituents, and (5) the search was restricted to employees in healthcare organizations. Papers were excluded if 1) the full text was not in the English language, 2) there was no measure of either employee voice or employee silence and 3) the sample did not consist of healthcare organization employees. The data retrieved from each database was logged in Excel and a duplicate control was conducted using EndNote. The remaining data was imported into Rayyan where two reviewers (OL and MKJ) had activated the blinding function in order to not see each other’s decisions, labels and notes.

**Figure 1:**
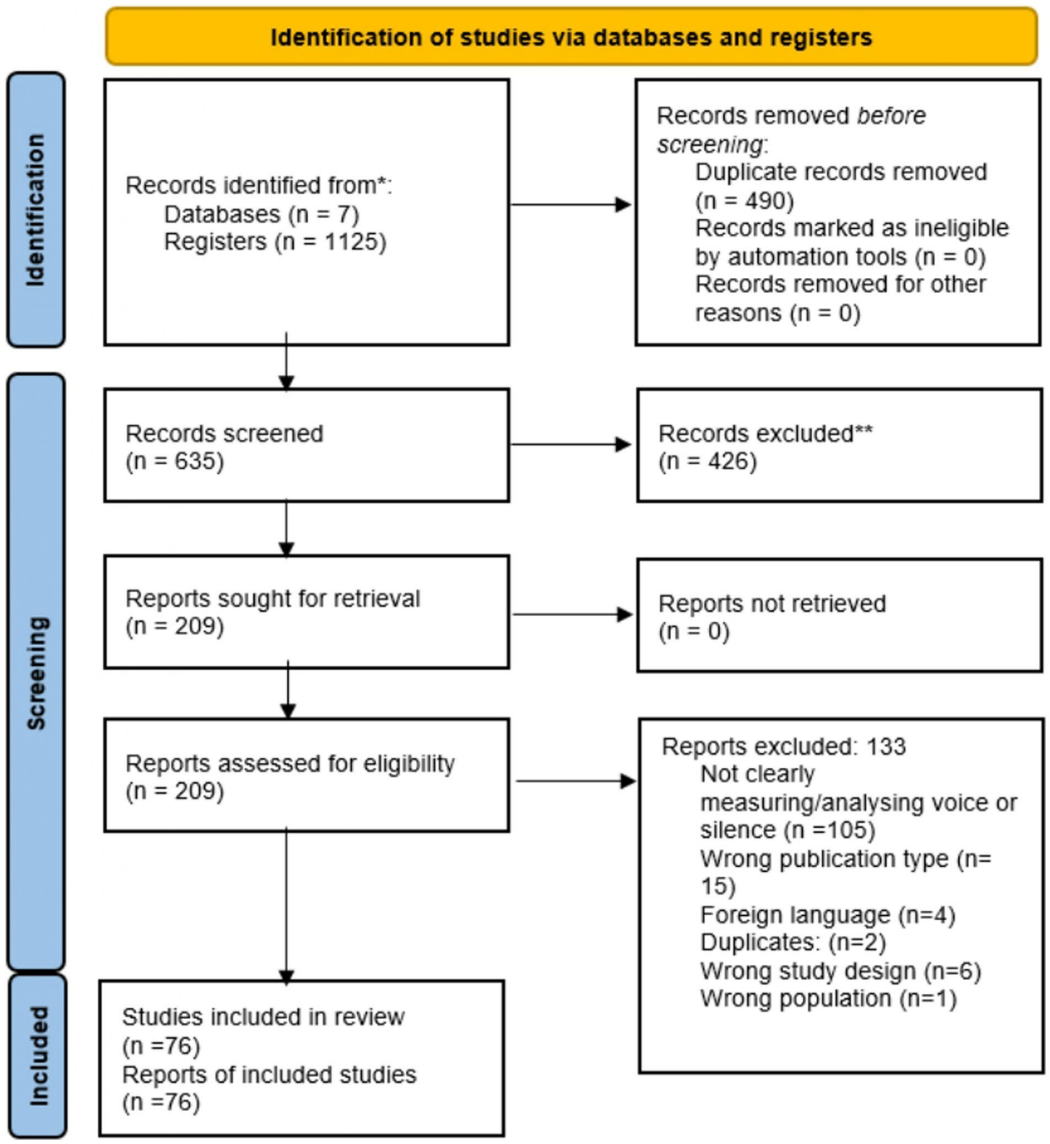
PRISMA flow diagram.

The results from the two independent reviewers (OL and MKJ) were compared (Cohen’s k = 0.96) and differences were discussed until an agreement was reached. A third reviewer was available when needed (AM). From the 209 studies that were screened in full text, a total of 76 papers met the criteria and were eligible for inclusion in the review. The PRISMA diagram below illustrates the number of papers identified, included and excluded throughout the process. In the first screening step, the abstracts were retrieved and read in order to decide which papers were relevant to include, based on the identified inclusion criteria. A full text review was conducted for the second screening step, using the same criteria, and the reasons for exclusion at this step are presented in the PRISMA diagram (see Figure 1). The most common reason for exclusion was that the study did not measure voice or silence.

Data were extracted from the 76 studies regarding the following information: Lead author and date; Study location; Professional group (e.g., nurses, physicians); Study design (e.g., cross-sectional/longitudinal; correlational/experimental); Sample size at baseline and follow-up; Female %; Measure of employee silence and/or voice outcome (see Table 1).

**Table 1:** Studies included in the review.

No	Article (Alphabetical)	Design	Sample (*n*)	(Total *N*, % Women)	Location	Measure of voice/speaking-up	Measure of silence
1.	Abd El-Fattah Mohamed Aly et al. (2021)	CS	Nurses (100%)	*N* = 322 F = missing	Egypt		Nurses silence Motives
2.	Abdelmotale et al. (2021)	CS	Nurses (100%)	*N* = 341 F = 68%	Egypt	Upward voice behavior scale (35)	
3.	Abdullah Mohamed et al. (2021)	CS	Nurses (100%)	*N* = 235 F = 88.9%	Egypt		Employee silence scale (36–38)
4.	Al-Abrrow (2018)	CS	Physicians (25.5%) Nurses (43.6%) Administrative staff (31.9%)	*N* = 346 F = 36.5%	Iraq		Employee silence scale (39)
5.	Alheet (2019)	CS	Physicians (25.6%) Nurses (15.4%) Pharmacists (13.8%) Other (45%)	*N* = 195 F = 29.74%	Jordan		Causative factors for organizational silence
6.	Alingh et al. (2018)	CS	Nurses (91.3%) Nurse managers (8.7%)	N = 980 Nurses *F* = 84.7% Nurse Managers *F* = 73.9%	Netherlands	Communication openness scale (40)	
7.	Amar et al. (2019)	CS					Employee silence scale (39)
8.	Amiri et al. (2018)	RCT Pre/post test	Nurses (78.7%) Supervisors (21.3%)	*N* = 61 F = 86.9%	Iran	Hospital Survey on Patient Safety Culture (AHRQ) (41)	
9.	Aslan et al. (2021)	CS	Nurse managers (100%)	*N* = 169 *F* = 81.1%	Turkey		Organizational silence scale (29)
10.	Avgar et al. (2016)	CS	Nurses (100%)	*N* = 363 F = 87%	United States	Employee patient-care voice (42)	
11.	Best and Kim (2019)	Pre and post intervention	Nurses (36.7%) Plastic surgery residents (22%) Internal medicine residents (41.3%)	*N* = 109 F = missing	United States	Speaking up intentions	
12.	Bilotta et al. (2021)	CS	Nurses (37%) Medical/dental (9%) *Health professionals or related healthcare staff (20%) Administrative/clerical (24%) Nonclinical managers (2%) Other occupational groups (9%)	*N* = 60,602 F = 80%	United Kingdom	Organizational level employee voice	
13.	Carpini (2020)	CS	Nurses (100%)	*N* = 46 F = 98%	Australia	Team behaviors scale	
14.	Çaylak and Altuntas (2017)	CS	Nurses (100%)	*N* = 323 F = 93.2%	Turkey		Employee silence scale (36–38)
15.	Chang et al. (2019)	CS	Nurses (100%)	*N* = 247 F = 96%	Taiwan	Voice behavior scale (43)	
16.	Darawad et al. (2020)	CS	Nurses (100%)	*N* = 233 *F* = 73.8%	Jordan	Vignettes—Speaking up Intention	
17.	De los Santos et al. (2020)	CS	Nurses (100%)	*N* = 549 F = 78.7%	Philippines		Employee silence scale (39)
18.	Doo and Choi (2020)	CS	Nurses (100%)	*N* = 301 F = 97.3%	South Korea		Employee silence scale (39)
19.	Doo and Kim (2020)	CS	Nurses (100%)	*N* = 301 F = 97.3%	South Korea		Employee silence scale (39)
20.	Erkutlu and Chafra (2018)	CS	Nurses (100%)	*N* = 913 F = 79%	Turkey		Employee silence scale (39)
21.	Gauld et al. (2020)	Longitudinal	2012 Allied/Other (34%) Physicians (19%) Midwife (3%) Nurses (44%) 2017 Allied/Other (29%) Physicians (20%) Midwife (4%) Nurses (46%) Missing = (1%)	2012 *N* = 10,303 F = 69% M = 20% Missing = 11% 2017 *N* = 8,541 F = 68% M = 19% Missing = 13%	New Zealand	Safety climate survey (44)	
22.	Ginsburg et al. (2017)	Pre and post intervention	(T1) pretest Nurses (62.5%) Allied (8.8%) Physicians (2.5%) (T2) posttest Nurses (64.1%) Allied (15.4%) Physicians (2.6%) (T3) posttest Nurses (73.7%) Allied (2.6%) Physicians (2.6%)	(T1) pretest *N* = 83 F = 85.5% (T2) posttest *N* = 39 *F* = 88.9% (T3) posttest *N* = 38 *F* = 86.5%	Canada	Teamwork climate survey (45)	
23.	Gong et al. 2021	Longitudinal (time-lagged)	Nurses (100%)	*N* = 608 *F* = 75%	China	Employee voice (46)	
24.	Gkorezis et al. (2016)	CS	Nurses (100%)	*N* = 157 *F* = 61.8%	Cyprus		Employee silence scale (47)
25.	Guo et al., 2021	CS	Nurses (100%)	*N* = 1717 *F* = 93.1%	China	Employee voice (46)	
26.	Gupta et al. (2018)	CS	Healthcare employees (100%)	*N* = 168 F = 61%	India	Prosocial voice (39)	
27.	Guris et al. (2019)	Prospective observational study with a nested double-blind randomised controlled components	Anesthesiology trainees (100%)	*N* = 22 *F* = 63.6%	United States	Observations of speaking up behaviors in simulations	
28.	Henkin et al. (2016)	Pre and post intervention	Before intervention Residents (51.8%) Nurses (22.7%) Attendings (25.5%) After intervention Residents (59%) Nurses (11.5%) Attendings (29.5%)	Before intervention *N* = 141 After intervention *N* = 122 F = missing	United States	Safety Attitudes Questionnaire (48)	
29.	Herrington and Hand (2018)	Pre-post intervention	Nurses (100%)	*N* = 26 F = missing	United States	Hospital Survey on Patient Safety Culture (AHRQ) (41)	
30.	Holland et al., 2017	CS	Nurses (89%) Midwives (9%) Personal carers (2%)	*N* = 1,036 *F* = 92%	Australia	Direct voice	
31.	Hu and Casey (2021)	CS	Healthcare workers without supervisory responsibilities (79.8%) Workers with some supervisory responsibility (12.5%) Principal or senior staff members with managerial responsibilities (7.7%)	*N* = 165 F = missing	Australia	Safety voice (49)	
32.	Islam et al. (2017)	CS	Nurses (100%)	*N* = 564 *F* = 76.4%	Pakistan	Voice behavior scale (43)	
33.	Jeong et al. (2021)	CS	Nurses (100%)	*N* = 120 *F* = 92.5%	Korea		Employee silence scale (47)
34.	Jungbauer et al. (2018)	CS	Physicians (25%) Nurses (55.4%) Other occupational groups with direct patient contact (10.2%)	*N* = 480 Missing = (9,4%) *F* = 67.1% M = 26.9% Missing = 6%	Germany	Incident reporting intention (50)	
35.	Kaya and Eskin Bacaksiz (2021)	CS	Nurses (100%)	*N* = 341 F = 80.4%	Turkey		Employee Silence Scale (Turkish Version) (39)
36.	Kesselheim et al. (2021)	CS	Pediatric Trainees (100%)	*N* = 233 *F* = 68%	United States	Vignettes—Speaking up Intention	
37.	Krenz et al. (2020)	CS	Nurses (46.1%) Residents (37.2%) Consultants (16.7%)	*N* = 78 *F* = 51%	Switzerland	Observations of Speaking Up behaviors in simulations	
38.	Kritsotakis et al. (2021)	CS	Nurses (100%)	*N* = 607 *F* = 83.5%	Greece		Employee Silence Scale (47)
39.	Labrague and De los Santos (2020)	CS	Nurses (100%)	*N* = 624 *F* = 80.3%	Philippines		Employee Silence Scale (39)
40.	Lawson et al. (2017)	CS	Perfusionists (100%)	*N* = 269 F = missing	United States	Hospital Survey on Patient Safety Culture (AHRQ) (41)	
41.	Lee and Dahinten (2021)	CS	Nurses (100%)	*N* = 526 *F* = 98.3%	South Korea	Speaking-up about Patient Safety	
42.	Lemke et al. (2021)	Observational study	Anesthesia care providers (100%)	*N* = 49 *F* = 46.9%	Switzerland	Observations of Speaking Up behaviors in simulations	
43.	Loewenbruck et al. (2016)	CS	Physicians (100%)	*N* = 251 *F* = 17.5%	Germany Japan United States	Vignette—Disclosure Intention	
44.	Luff et al. (2021)	CS	Radiology trainees (100%)	*N* = 61 *F* = 43%	United States	Speaking-up about patient-safety concerns and unprofessional behaviors (51)	
45.	MacMahon et al. (2018)	CS	Nurses (100%)	*N* = 2,929 *F* = 96.6%	Ireland		Antecedents of not reporting bullying (52, 53)
46.	Manapragada and Bruk-Lee (2016)	CS	Employees from healthcare (76%) Employees from construction/utility (12%) Employees from retail (12%)	*N* = 311 F = 98%	United States		Safety Silence Motives
47.	Mansour et al. (2020)	CS	Nurses (100%)	*N* = 83 F = 88%	Saudi Arabia	Speaking-up Scale (54)	
48.	Martinez et al. (2016)	CS	Postgraduate trainees internal medicine (58%) Postgraduate trainees surgery (42%)	*N* = 352 *F* = 49%	United States	Speaking-up about Patient Safety (51)	
49.	Martinez et al. (2017)	CS	Postgraduate trainees internal medicine (58%) Postgraduate trainees surgery (42%)	*N* = 837 *F* = 50%	United States	Speaking-up about Patient Safety and Unprofessional Behavior (51)	
50.	Mesdaghinia et al. (2021)	CS	Healthcare employees (100%)	*N* = 134 F = 76%	United States	Prohibitive Voice (46)	
51.	Mousa et al. (2021)	CS	Physicians (100%)	*N* = 229 *F* = 18.8%	Egypt		Employee Silence Scale (55)
52.	Ng et al. (2017)	CS	Physicians (18.8%) Nurses (81.2%)	*N* = 80 *F* = 71.2%	China	Communication Openness (41)	
53.	Noviyanti et al. (2021)	CS	Nurses (100%)	*N* = 51 *F* = 76.5%	Indonesia	Hospital Survey on Patient Safety Culture (AHRQ) (41)	
54.	Oner et al. (2018)	RCT	Nurses (100%)	*N* = 70 *F* = 100%	United States	Observations of speaking up behaviors in simulations	
55.	Ortiz-Lopez et al. (2021)	CS	Physicians (42.4%) Non-medical profession (Nurses, midwifes among others) (57.6%)	*N* = 203 F = 63%	Chile	Attitudes towards speaking up	
56.	Ozyilmaz and Taner (2018)	CS	Physicians (15.7%) Dentists (6%) Nurses (45.2%) Psychologists (1.7%) Nutritionists (1.3%) Delivery Nurse (4.3%) Pharmacists (1%) Technicians (18.1%) Biologists (1%) Other (5.7%)	*N* = 299 *F* = 65.6%	Turkey	Voice behavior scale (43)	
57.	Parlar-Kilic et al. (2021)	CS	Nurses (100%)	*N* = 671 F = 80.3%	Turkey		Organizational Silence Scale (56–58)
58.	Polat et al. (2018)	CS	Nurses (100%)	*N* = 329 *F* = 95.7%	Turkey		Organizational Silence Scale (56–58)
59.	Rainer and Schneider (2020)	CS	Nurses (100%)	*N* = 303 F = 93%	United States	Safety Attitudes Questionnaire (48)	
60.	Raemer et al. (2015)	Simulation-based randomized controlled experiment	Practicing non-trainee Anesthesiologists (100%)	*N* = 340 F = missing	United States	Observations of speaking up behaviors in simulations	
61.	Reyhanoglu and Akin (2020)	CS	Nurses (43.3%) Medical assistants (15.3%) Medical clerks (27%) Health technicians (14.4%)	*N* = 367 *F* = 68.1%	Turkey		Employee silence scale (39)
62.	Richard et al. (2021)	CS	Healthcare professionals (nurses and physicians) (100%)	*N* = 523 F = 83%	Switzerland	Speaking-up about patient safety	
63.	Ridley et al. (2021)	Pre and post intervention	Healthcare professionals (100%)	Baseline *N* = 73 Follow up 6 months *N* = 68 Follow up 12 months *N* = 68 F = missing	United States	Hospital Survey on Patient Safety Culture (AHRQ) (41)	
64.	Roussin et al. (2018)	CS Observational Simulation Study	Nurses Physicians	*N* = 129 *F* = 53.8%	Spain	Speaking up behavior	
65.	Schwappach (2018)	CS	Nurses (77.7%) Residents (9%) Doctors (13.4%)	*N* = 1,217 *F* = 78.3%	Switzerland	Speaking-up about patient safety	
66.	Schwappach and Niederhauser (2019)	CS	Nurses (50.3%) Physicians (11.6%) Psychologists (12.7%) Other (20%) Missing (5.4%)	*N* = 817 *F* = 67.68	Switzerland	Speaking-up about patient safety	
67.	Schwappach and Richard (2018)	CS	Nurses (79%) Physicians (21%)	*N* = 979 F = 81%	Switzerland	Speaking-up about patient safety	
68.	Schwappach and Sendlhofer (2019)	CS	Nurses (81%) Physicians (16.5%) Missin (2.5%)	*N* = 768 *F* = 74%	Switzerland	Speaking-up about patient safety	
69.	Schwappach et al. (2018)	CS	Nurses (72.8%) Physicians (15.2%) Other (8.4%) Missing (3.6%)	*N* = 859 *F* = 74.6%	Austria	Speaking-up about patient safety	
70.	Seren et al. (2018)	CS	Nurses (87.9%) Physicians (12.1%)	*N* = 601 *F* = 90%	Turkey		Employee Silence Scale (56–58)
71.	Toy et al. (2019)	CS	Anesthesiology trainees (74.1%) Anesthesiologists (25.9%)	*N* = 81 *F* = 44.44%	United States	Vignettes—speaking up intention	
72.	Voogt et al. (2019)	CS	Residents (100%)	*N* = 299 *F* = 70%	Netherlands	Voice behavior scale (43)	
73.	Weiss et al. (2017)	Pre and post intervention	Resident anesthesiologists (50%) Anesthesia nurses (50%)	*N* = 40 *F* = 67.5%	Switzerland	Observations of speaking up behaviors in simulations	
74.	Yalcin and Baykal (2019)	CS	Nurses (62.4%) Physicians (29.1%) Physiotherapists (3.7%) Dieticians (2.6%) Emergency medical technicians (2.2%)	*N* = 463 F = missing	Turkey		Silence Motives
75.	Zhang et al., 2021	CS	Nurses (100%)	*N* = 1,221 *F* = 92.79%)	China	Employee voice (46)	
76.	Zhou et al. (2021)	CS	Nurses (100%)	*N* = 598 *F* = 94.6%	China	Employee voice (46)	

### 2.4. Quality appraisal

Quality assessment was conducted using the Quality Assessment Tool for Observational Cohort and Cross-Sectional Studies (59). Three reviewers independently assessed the quality of the included studies (CM, KP and CC) and a fourth reviewer was invited in case of disagreement (OL or AM). The tool contains 14 criteria, and the evaluator is asked to answer whether the study in question meets the criterion, with the possible answers being “Yes, No, Cannot Determine, Not-applicable, and Not Reported”. A score of >/=11 corresponds to good quality, 7–10 to fair quality and < 7 to poor quality. As this was an Integrative Systematic Review employing a narrative synthesis, risk of bias assessment by generating a synthesized result (e.g. a meta-analytic effect estimate, or median effect across studies) was not possible, meaning that the process is more conducive to systematic reviews/meta-analyses in which the sampling frame is narrow, the research designs included are similar or even identical and outcomes assessed are not fundamentally heterogeneous (32).

### 2.5. Data analysis and interpretation

As this integrative review looked more broadly at the phenomenon of employee silence and voice in healthcare, with the aim of creating clarifications and insights, a holistic approach was sought. No papers were excluded based on quality assessment results, even though such a quality assessment was conducted. We summarized the data extraction results using descriptive statistics.

To supplement the findings regarding the measures of employee voice and silence in healthcare, thematic analysis of the relevant measures of each study was used to identify themes with constant comparative analysis (47, 60). A constant comparison method is a widely used approach used that allows the conversion of data into systematic categories, which allows the researcher to identify distinct themes or variations. In IRs, this approach is compatible with the use of varied data from diverse methodologies (32). The coding process was inductive, meaning that there were no pre-defined codes and an extensive review of the contents of each measure was conducted to develop initial codes. It is a common challenge in qualitative analysis that initial coding leads to an overwhelming number of shallow codes (47). Discussion among three authors (OL, MKJ and AM) helped develop the analysis throughout the process as multiple instances of codes occurred in close proximity to each other highlighting potential connections between codes. Each study was independently analyzed and coded by OL in conjunction with one other independent reviewer (MKJ, AM, CM, KP). The coding process was reviewed until agreement was reached regarding the most meaningful criteria and categories of analysis emerging from the process. In a process of constant comparison, extracted data on measures were compared item by item so that similar data were categorized and grouped together, and these coded categories were then compared to further the analysis and synthesis (32). OL collated all proposed codes, which the research team collectively discussed to create a comprehensive and shared understanding of each code. Due to the diverse representation of employee voice and silence measures, these were coded using constant comparative analysis according to the following criteria: a) context, meaning whether the measures were patient-safety specific or aimed at measuring employee silence/voice in a general context; b) conceptual distinction, meaning whether data on employee silence (withholding voice) or employee voice (speaking up) were collected; and c) aspects, meaning what aspects of employee silence and/or voice/speaking up the measures aimed to capture. Based on the first criterion (a) two meaningful categories were coded as 1) patient-safety specific and 2) general context. For the second criterion (b), measures were coded as 1) measures of employee silence, 2) measures of employee voice/speaking up and 3) measures of multiple aspects of employee silence and employee voice/speaking up. For the third criterion (c), measures were coded based on what specific aspect of employee silence/voice they were aiming to capture; examples include: antecedents of silence (i.e., motives and content); intention to speak up; perceptions of speaking-up related climate; self-reported past speaking up behavior; externally observed speaking-up behaviors. All authors reviewed and agreed on the themes’ codes from the data analysis. The themes and subthemes are presented in Table 2.

**Table 2:** Categorization of studies according to context, conceptual distinction, and aspects of employee voice/silence.

Criterion A	Criterion B	Criterion C	*N* of distinct measures
1. Patient Safety	1. Silence	- Patient Safety Employee Silence Antecedents (i.e., self-reported frequency of having remained silent about patient safety issues due to specific reasons or regarding specific content/in specific situations)	3
	2.Voice/speaking up	- Likelihood/intentions of Speaking-up regarding patient safety (i.e., self-reported likelihood of speaking-up regarding patient safety)	6
		- Speaking-up for Patient Safety Climate (i.e., perceptions regarding how speaking-up for patient safety is regarded in the team/organization)	10
		- Externally observed Speaking-up behaviors (i.e., externally observed speaking up behaviors in training simulations based on predefined criteria)	7
	3.Multiple aspects of employee silence/voice	- Multiple aspects of patient-safety silence/voice (e.g., antecedents of patient safety silence and speaking up intentions)	4
2. General context	1. Silence	- Employee Silence Antecedents and Consequences (i.e., self-reported frequency of having remained silent due to specific reasons or regarding specific content/in specific situations)	7
	2.Voice/speaking up	- Employee Voice/Speaking Up Antecedents (i.e., self-reported speaking-up behavior due to specific reasons or regarding specific content/in specific situations)	2
		- Employee voice/speaking-up self-reported behavior (i.e., self-reported frequency of having spoken up)	2
		- Speaking-up related climate (i.e., perceptions regarding how speaking-up is regarded in the team/organization)	4

Criterion A: context, meaning whether the measures were patient-safety specific or aimed at measuring employee silence/voice in a general context.

Criterion B: conceptual distinction, meaning whether data on employee silence (withholding voice) or employee voice (speaking up) were collected.

Criterion C: aspects, meaning what aspects of employee silence and/or voice/speaking up the measures aimed to capture.

## 3. Results

### 3.1. Sample

The total sample of participants was 122,009 (N = 122,009, 69.3% female), while 13% of the included studies did not report information of the participants’ gender (40, 54, 61–65)). Of the total sample, 57,520 were identified as nurses (47.1%); 11,228 (9.2%) were nurse managers/ supervisors/ head nurses; 1736 (1.4%) were nurse experts; 9,266 (7.6%) were physicians; 4,279 (3.5%) were residents or trainees; and 392 (0.32%) were senior physicians. Moreover, 18,468 (15.1%) were allied health professionals (e.g., physiotherapists, psychologists, dieticians etc.); 5,792 (4.7%) participants were classified as “other” and 132 (0.11%) as administrative staff. Seven studies had unspecified samples (n = 2,609; 2.1%): Avgar et al. (49) with 363 unspecified healthcare workers; Gupta and Ravindranath (66) with 1700 unspecified eye hospital employees; Hu and Casey (67) with 165 unspecified health care workers; Lawson et al. (64) did not report sample size of participants (perfusionists); Lemke et al. (68) did not specify on their sample of 49 anesthesia care providers; Mesdaghinia et al. (69) reported a sample of 203 supervisors in a hospital; finally, Roussin et al. (70) did not provide the numbers of nurses and physicians in their sample (*n* = 129). Thus, based on the information provided, most of the participants were nurses (57.7% cumulative percentage of nurses, nurse managers and nurse experts).

Detailed information on departments/units was available only for 13.5% of the total sample. Based on the information reported in the studies, 7.2% of the participants worked in surgical departments; 2.9% in Anesthesiology; < 1% in ICUs; 1.4% Eye Hospital; 1.1% in Internal Medicine; 0.4% in Emergency Departments, followed by Pediatrics (0.3%); Cardiology (0.2%); Radiology (> 0.1); OB/Gyn (0.1%); Nephrology (0.07%); Gastroenterology (0.07%); Orthopedics (0.07%); OR (0.03%); Outpatient Units (0.02%); Plastic Surgery (0.02%).

In terms of location, 21 studies were conducted in United States (26.9%)—one study was conducted in both Japan and the United States (1.3%)—10 studies were conducted in Turkey (12.8%), eight in Switzerland (10.3%), five in China (6.4%), four in Egypt (5.1%), three in South Korea (3.5%), three in Australia (3.5%) and three in the Philippines (3.5%); in two studies, participants were recruited form the Netherlands (2.6%) and in four studies participants were recruited from Germany (5.1%). Two studies were conducted in Jordan (2.6%). One study (1.3%) was conducted in each of the following countries: Austria; Spain; Indonesia; Saudi Arabia; Ireland; Greece; Pakistan; Cyprus; Canada; New Zealand; Taiwan; India; UK; Iraq; Iran. Thus, the most represented country was the United States with 21 studies (26.9%); however, it was noteworthy that 47.8% of the studies included in the current IR were conducted in countries outside of Europe, the United States and North America.

In terms of publication year, 19 studies were published in 2021 (24.4%), 14 studies in 2020 (17.9%), 10 studies in 2019 (12.8%), 15 in 2018 (19.2%), 12 in 2017 (14.5%), nine in 2016 (11.5%).

### 3.2. Quality appraisal

Of the 76 research reports that were included in the current integrative review, 8 (10.5%) were rated by three independent evaluators as good, 36 as fair (47.4%) and 32 studies (42.1%) were rated as having poor quality on the Quality Assessment Tool for Observational Cohort and Cross-Sectional Studies. This is strongly linked to the fact that the studies were predominantly cross-sectional, did not provide justification for the sample size (e.g., prospective power analysis) and did not always control for the effect of important relevant variables (e.g., confounders).

### 3.3. Measures

In total, 45 distinct measures of employee silence and employee voice/speaking up were identified across the 76 studies included in this IR. Thirty of these measures were identified as safety-specific, of which three were measures of employee silence, 23 were measures of employee voice/speaking up and four were measures including multiple aspects of patient safety-related silence and/or voice/speaking up. Of the remaining 15 general context measures, seven were measures of employee silence and 8 were measures of employee voice/speaking up. Examples of items for each measure are included in Table 3. Employee silence was measured in 31(40.8%) of the 76 studies and employee voice was measured in 53 (69.7%) out of the 76 studies, as some studies measured both voice and silence together.

**Table 3:** List of employee silence and voice measures.

	Measure	Example Items	Study/Studies
*Patient safety silence antecedents*	Silence about patient safety—Motives	‘Remaining silent to avoid encountering and fighting with his\her nursing colleagues and/or supervisors”	(18, 48, 61, 65, 71)
	Safety Silence Motives	“(I do not speak up to my supervisor when) … I feel that it could lead to a negative perception of me”	
	Employee Silence Scale (12)	“I kept quiet instead of asking questions when I wanted to get more information about patient safety in my department”	
*Intentions/Likelihood of speaking-up about patient safety*	Speaking-up Scale (72)	“On the night shift, you are working with another senior staff nurse who is a very good friend of yours. You have witnessed her administering the wrong dose of IV^1^ co-Amoxiclav^2™^ 1.2 g to the wrong patient, but it was too late for you to alert her. However, she told you that “it was only one dose, and the patient will be fine”. What would you do?”	(45)	
	Likelihood of Speaking up (73)	On the night shift, you are working with a senior staff nurse who is a very good friend of yours. You have witnessed her administering the wrong dose of intravenous co-amoxiclav 1,200 mg to the wrong patient, but it was too late for you to aler ther. However, she told you that ‘it was only oned ose, and the patient will be fine’.How likely are you to ask her to report this? Please circle the answer that best corresponds to your response (1 = very unlikely, 5 = very likely)	(73)	
	Disclosure Intention (51)	Case 1 (minimal harm to the patient) Case 2 (serious harm to the patient) “I would: (1) not disclose the adverse outcome (2) only disclose if it was caused by a mistake (3) disclose it even if it was not caused by a mistake.	(51)	
	Individual Speaking up Attitudes (74)	“I speak up if I see something that may negatively affect patient care.”	(44)	
	Voice subscale from the Safety Citizenship Behavior Scale (42)	“I speak up and encourage others to get involved in safety issues.”	(67)	
	Likelihood of Speaking up (*ad-hoc* questionnaire)	“When I encounter patient safety concerns, I am likely to speak-up to a team member/ to the person who is in a position of power and influence”.	(75)
*Perceptions of speaking-up about patient safety climate*	Hospital Survey on Patient Safety Culture—AHRQ (41)	“When staff in this unit see someone with more authority doing something unsafe for patients, they speak up.”	(50, 63, 64, 76–78)	
	“Safety Attitudes Questionnaire” (79)	“In this clinical area, it is not difficult to speak up if I perceive a problem with patient care.”	(62, 80, 81)	
	Teamwork Climate Survey (82)	“In this unit, it is difficult to speak up if I perceive a problem with patient care.”	(83)	
	Safety Communication Scale (39)	“I feel comfortable discussing safety issues with my supervisor.”	(65)	
	Safety Climate Survey (84)	“In this clinical area, it is easy to speak up if I perceive a problem with patient care.”	(85)	
	Perception of speaking-up based on Communication Oppenness (86)	(The extent to which ICU members within a group can speak openly without fear of negative repercussions/ misunderstandings)	(87)	
	Speaking up Climate for Safety Scale (88)	“In my clinical area it is difficult to speak up if I have a patient safety concern.”	(80)	
	Speaking up Climate for Professionalism Scale (88)	“In my clinical area it is difficult to speak up if I observe unprofessional behavior.”	(80)	
	Employee Patient-Care Voice (89),	“Supervisors ask for the employees’ input regarding important patient care issues.”	(49)	
	Reporting-specific trust (90)	“I trust that there will be no disadvantages for my future career if I report an adverse event.”	(91)
*Externally observed speaking-up behaviors (Patient safety specific)*	Advocacy-Inquiry rubric (modified)	Use of advocacy and inquiry language Timing	(92)	
	Co-ACT coding system (modified)	Utterances involving either suggestion-, problem-, opinion-, or doubt-focused content Frequency of voice occurrences Time to voice	(93, 94)	
	An observation system based on both organizational behavior and anaesthesia research	Referring to medication, airway, and/or anaesthesia induction procedure: “It’s better to make a rapid sequence induction for this patient” Stating an opinion or point of view: “I have doubts about a 200 mg bolus of Propofol, because the patient has a left ventricular ejection fraction of 30%”	(68)	
	Five-point modified Pian-Smith (95) grading scale	1 = saying nothing, showing no expression 5 = advocates or inquires repeatedly, initiates discussion	(96)	
	Speaking-up during scripted opportunities	Specific events presenting with particular issues (e.g., profound sleepiness)	(92, 97)	
	Assessment based on predefined desired actions for each event	e.g., Whether the participant asked the surgeon if he/she wanted help and/or asked the circulating nurse to get help for the surgeon.	(97) (94)	
	Frequency of observed behaviors that encourage speaking up	Restate Ask open questions Clarify	(54)
*Scales measuring multiple aspects of patient-safety silence/voice*	“Speaking Up about Patient Safety Questionnaire”	Safety Silence: “How often did you choose not to bring up your specific concerns about patient safety?” Speak-up about safety: “How often did you bring up specific concerns about patient safety?” Likelihood of speaking up: “How likely is it that you try to alert the consultant to the missed hand disinfection/gloves using words or gestures?”	(55, 78, 98–100)	
	Speaking up about medical errors	*Attitudes towards speaking up*: “Communication of errors between professionals strengthens the performance of the health team.” *Perceptions of speaking-up related climate*: “It is frowned upon in the work-environment that the acting physician is questioned.” *Intention to speak up*: “I will take advantage of the meeting instances that exist in our health centre to communicate my opinion on clinical practices. *Past speaking up behavior*: “I have mentioned specific concerns about patient safety.”	(101)	
	Speaking-up about patient safety	*Past speaking-up behaviors*: Did you discuss any of the unprofessional behaviours or patient safety breaches they observed with the person(s) involved? *Speaking up climate*: “In my clinical area, it is difficult to speak up if I observe a safety breach” *Antecedents of speaking up*: “Fear of conflict or eliciting anger” *The likelihood of speaking up*: two hypothetical scenarios for assessing the likelihood of speaking up about traditional and professionalism-related patient safety threats	(80, 102)	
	Speaking-up about patient safety	*Likelihood of speaking-up*: three hypothetical scenarios *Speaking-up antecedents*: “My team will view me as competent if I effectively express patient safety concerns in the OR”	(52, 92)
*Employee Silence Antecedents and Consequences—General context*	*Employee Silence (* *53* *)*	*Prosocial silence: “…withholding confidential information based on cooperation.”* *Acquiescent silence: “…be unwilling to speak up with suggestions for change because they are disengaged.”* *Defensive silence “…do not speak up and suggest ideas for change based on fear.”*	*(**17*, *36–38*, *40*, *103–108**)*	
	Employee silence (40)	Silence Climate Silence based on fear Acquiescence Silence Silence based on protecting the organization	(108)	
	Employee silence (43)	“I practice organizational silence for the following reasons…” “…low trust in administrators and managers” “…receiving negative views on me”	(43)	
	Employee Silence—superior/subordinate relationship (109)	“…fear of confrontation or avoiding arguments” “…lack of interest in taking responsibility or ownership”	(110)	
	Antecedents of silence about bullying (11, 111)	“I feel it would negatively affect my career” “Complaints are actively discouraged”	(112)	
	Antecedents and consequences of employee silence (46)	“How often do you express your disagreements to your managers concerning your departments’ issues?”	(65)	
	Antecedents and consequences of employee silence (56–58)	*Motives*: administrative and organizational issues; work issues; lack of experience; fear of isolation; and fear of damaging relationships *Content*: ethics and responsibilities; administrative problems; employees’ performance; improvement efforts; and working facilities. *Consequences*: consequences that prevent performance and synergy; consequences that limit improvement and development; and consequences that upset personnel	(35, 113–116)
*Employee Voice Antecedents—General context*	Voice Behavior Scale (117)	Promotive voice: “I develop and make recommendations concerning issues that affect my work group”	(66, 107, 118–121)
	Employee Voice (122)	*Promotive Voice:* “Proactively develop and make suggestions for issues that may influence the unit.” *Prohibitive Voice:* “Speak up honestly with problems that might cause serious loss to the work unit, even when/though dissenting opinions exist.”	(69, 123–126)
*Employee voice/speaking up self-reported behavior*	*Upward Voice Behavior (* *35* *)*	*“I give my supervisor constructive suggestions regarding work-related issues.”*	(127)
	*Ad-hoc* questionnaire	“I spoke up in this course when I was unclear about something that the faculty had explained.”	(70)
*Perceptions of Speaking-up Related Climate*	*Organizational-level employee voice (ad-hoc questionnaire)*	*“I am able to make suggestions to improve the work of my team/department.”*	(128)
	Team Behaviors Scale	The extent to which they observed proactive speaking-up behaviors	(129)
	Direct Voice	Existence of formal voice channels: regular meeting between senior management and all staff; formal employee involvement program; and semi-autonomous workgroups	(130)
	*Ad-hoc* questionnaire	“I felt comfortable asking questions and expressing concerns to other team member.”	(77)

#### 3.3.1. Patient-safety specific measures

The majority of distinct measures—30 out of the 45—identified across the studies included in the current review were safety specific. This category includes measures that are focused on safety issues.

##### 3.3.1.1. Patient safety employee silence antecedents

Three distinct measures were identified in this category across five studies. All three measures aimed at capturing antecedents of employee silence regarding patient safety issues (e.g., motives). Two of these measures were developed to measure motives for patient-safety silence and each was used in one study, and one for patient-safety related silence in specific situations/regarding specific content.

A scale for self-reported frequency of remaining silent about patient safety due to specific reasons was developed and tested by Abd El-Fattah Mohamed Any et al. (61), examining the following motives: avoidance, belief, attitude, fear, management and organization.

The “Safety Silence Motives” scale was developed and tested by Manapragada and Bruk-Lee (65) to measure frequency of remaining silent for reasons that are relationship based, climate-based, issue-based and job-based motives. Both (61, 65) were validation studies.

The third measure in this category is the scale adapted by Tangirala and Ramanujam (12) and was used in three (3.9%) of the 76 studies (18, 48, 71).

##### 3.3.1.2. Likelihood/ intentions of speaking-up regarding patient safety

Six distinct measures were identified in this category across seven studies. The measures in this category aimed at capturing the likelihood/intentions of speaking up about patient safety.

Three of the measures included in this category were questionnaires that used hypothetical scenarios (vignettes) related to patient safety issues. These include: the Speaking-up Scale developed by Andrew and Mansour (72) which was used in the study of Mansour et al. (45); a questionnaire with four hypothetical scenarios (73); and the study of Lowenbruck et al. (51), evaluating participants’ disclosure intention using two hypothetical scenarios with increasing adverse outcome severity.

The remaining three measures in this category were scales asking the participants to rate their speaking-up intentions. The Individual Speaking up Attitudes was used by Alingh et al. (44), which was an adaptation of the Communication Openness Scale (74). The voice subscale (four items) from the Safety Citizenship Behavior Scale (42) was used by Hu and Casey (67) to measure Safety Voice. Although the scale refers to safety in general (not specifically for healthcare/patient), it is included in the patient-safety specific measures, as in healthcare any reference to safety is always linked to patient safety. Best and Kim (75) measured participants’ likelihood of speaking up about patient safety pre- and post-intervention using two items (towards a team member and towards persons who are capable of change).

##### 3.3.1.3. Speaking-up about patient safety climate

The measures in this category include items that aimed at capturing the participants perceptions regarding speaking-up-related climate for patient safety issues. Ten distinct measures were identified in this category across 16 studies.

The Hospital Survey on Patient Safety Culture (HSOPSC) developed by the Agency for Healthcare Research and Quality (AHRQ) (41) was used in six (7.9%) of the 76 studies (50, 63, 64, 76–78). The survey includes four items related to speaking up about patient safety in the “Communication” subscale.

The Safety Attitudes Questionnaire (SAQ) (79) was used in three studies (3.9%) (62, 80, 81) which includes items relevant to speaking-up in the Teamwork Climate and the Safety Climate subscales.

Each of the remaining eight measures was used only in one study. In particular, a modified version of the Teamwork Climate Survey (82) was used by Ginsburg and Bain (83). The Speaking up Climate for Safety Scale (88) and the Speaking up Climate for Professionalism Scale (88) were used in the study of Martinez et al. (80).

One item related to the speaking-up climate from the Safety Climate Survey (84) was used in the study of Gauld and Horsburgh (85). Avgar et al. (49) measured Employee Patient-Care voice using two items adapted from Clark et al. (89), assessing the degree to which employees perceived that their input regarding important patient care issues is taken into account. Ng et al. (87) measured the participants perceptions of speaking-up using a scale developed to measure communication openness based on the work of Reader et al. (86), consisting of separate questions for nurses and doctors. Jungbauer et al. (91) included modified items from the work of Wu et al. (90), to measure “reporting-specific trust” which are similar to those used in other scales to measure perceptions of speaking up related climate.

The Safety Communication Scale (39) was used in one study (65) to assess how comfortable participants felt in sharing their concerns and ideas regarding safety. Although the scale refers to safety in general (not specifically for healthcare/patient safety), it is included in the patient safety specific measures, as for similar reasons to Hu and Casey’s (67) Safety Voice mentioned above.

##### 3.3.1.4. Externally observed speaking-up behaviors

This category includes measures that aimed at capturing aspects of externally observed speaking up/voice behaviors. The following seven distinct assessments were used to rate externally-observed speaking-up behaviors across seven observational studies: the Advocacy-Inquiry rubric in the study of Guris et al. (92); the Co-ACT coding system in two studies (93, 94); an observation system based on both organizational behavior and anesthesia research (68); the five-point modified Pian-Smith (95) grading scale ranging from 1 being silence to 5 being repeated inquiry (96); level of speaking-up during the scripted opportunities when speaking up was anticipated in two studies (92, 97); assessment based on predefined desired actions for each event (97); and frequency of observed behaviors that were identified through focus groups as encouraging speaking up (e.g., restate; ask open questions; clarify, etc.) (65).

These seven observational studies (9.2% of the 76 studies)—meaning that speaking up/voice was measured by observing participants’ speaking-up behaviors—also discussed the following aspects of speaking-up: voicing frequency (68, 93), the level of assertiveness (68, 96), content (e.g., patient-safety concern, innovative ideas) (68, 93), and who the participants spoke-up to (93, 97). In some cases, other aspects were taken into account as well, such as clinical relevance (68), whether speaking up was prompted or unprompted (92), quality (92), and time to voice (93). One study was focused on the observation of behaviors encouraging speaking up rather than monitoring actual speaking-up behaviors (54).

##### 3.3.1.5. Scales measuring multiple aspects of patient-safety silence/voice

This category includes four measures that aimed at capturing multiple aspects of silence and/or voice/speaking up included in one questionnaire. There four measures were identified across 11 studies.

The first measure in this category is the Speaking Up about Patient Safety Questionnaire that was used in six (7.9%) of the 76 studies included in this IR (55, 78, 98–100, 131). The measure includes one subscale that measures the frequency of having remained silent regarding specific content, conceptualized as withholding information about patient safety; one subscale that measures the frequency of having spoken up regarding specific content; and four questions measuring the likelihood of speaking up in a hypothetical situation.

Martinez et al. (80) used a questionnaire related to multiple aspects speaking up behavior, including past speaking-up behaviors; antecedents of speaking up about patient safety or unprofessional behavior (facilitators and barriers); speaking-up related climate and the likelihood of speaking up towards different team members (e.g., nurse, intern) with regard to two scenarios. Kesselheim et al. (102) also used this questionnaire.

Ortiz-Lopez et al. (101) developed and tested a scale focused on speaking up about medical errors, including subscales for attitudes towards speaking up; perceptions of speaking-up related climate; intention to speak up; and past speaking up behavior.

Toy et al. (52) used a questionnaire that measured the likelihood of speaking-up conceptualized as assertive communication, with three hypothetical scenarios related to patient safety concerns and 10 items measuring speaking-up antecedents, including intrapersonal factors that might influence speaking-up specifically for the operating room. The latter was also used in the study of Guris et al. (92). An interesting aspect of the latter is that the authors included potential positive outcomes of speaking up (e.g., “Speaking up in the OR will increase my colleagues’ respect of my patient care skills”) as opposed to negative outcomes which is more common in voice/silence scales.

#### 3.3.2. General context measures

##### 3.3.2.1. Employee silence antecedents and consequences

Seven distinct measures were identified in this category. Five of these measures aimed at only measuring antecedents of employee silence, while the remaining two measures aimed at capturing antecedents and consequences of employee silence.

The most frequently used scale to measure employee silence motives in this category was the scale developed by Van Dyne et al. (53), which appeared in 9 (11.8%) out of the 76 studies included in this IR (17, 36–38, 103–107). The Van Dyne et al. (53) scale measures three types of employee silence based on three distinct motives: prosocial silence, acquiescent silence, and defensive silence. Al-Abrrow (103) re-labelled two of the three types of silence: quiescent silence (instead of acquiescent) and positive social silence (instead of prosocial silence).

The Yalcin and Baykal (40) scale for employee silence antecedents was used in two studies (2.6% of the 76 studies) (40, 108). This scale required the participants to indicate the frequency of remaining silent due to reasons related to silence climate, silence based on fear, acquiescent silence and silence based on protecting the organization. Though the scale was tested for use among healthcare professionals, it is included in the general context category as the items do not refer to patient safety specific silence.

The third measure in this category was developed by Alheet (43). The *ad-hoc* questionnaire purports to measure causative factors in the following dimensions: management and organization; experience; anxiety and fear; and being afraid of alienation. Though the scale is initially presented to be a multidimensional measure of causative factors, the authors analyze it as a unidimensional variable.

The fourth measure in this category was that developed by Jain (109), which was originally constructed to investigate dimensions of employee silence in the Indian work settings and specifically focusing on the supervisor-subordinate relationship; the scale was used by Mousa et al. (110) to measures reasons for employee silence with respect to their supervisors, organized in four factors: fear of retaliation; internal motivation; self-competence; self-image. The model was treated as a one-factor-model in this study (110).

The fifth measure in this category was developed by MacMahon et al. (112) to assess bullying reporting antecedents and was based on the work of Pinder and Harlos (11) on the antecedents of employee silence, as well as the work of Whiteside and Barclay (111).

As mentioned earlier, two scales aimed at measuring both antecedents and consequences of employee silence. The scale developed by Vakola and Bouradas (46) was used in one study (65). The last scale in this category—and the second to measure both antecedents and consequences—was the employee silence scale developed by Cakici (56–58) that was used in five (6.6%) of the 76 studies included in this IR (35, 113–116). Antecedents include items related to both motives and content. The consequences subscale was used only by Seren et al. (117). Caylak and Atluntas (114) only measured the antecedents and the other three studies (35, 113, 115) only measured the frequency of silence motives.

##### 3.3.2.2. Employee voice/speaking up antecedents

Two distinct measures of employee voice antecedents (e.g., motives, content) were identified across 12 studies out of the 76 included in this review.

The Voice Behavior Scale by Van Dyne and Lepine (117) was used in six (7.9%) out of the 76 studies (56, 66, 107, 119–121). Items refer to speaking up in the team as a promotive behavior—also defined as prosocial or promotive voice (30)—and are based on the approach that views voice as one of the helping and/or extra role behaviors (117).

The employee voice scale developed by Liang et al. (122) was used in six (7.9%) of the 76 studies (69, 123–126). This scale measures promotive voice and prohibitive voice where the former refers to “putting forward new ideas and methods to improve the efficiency of the enterprises” and the latter refers to “expressing the inhibitive viewpoint and the harmful problem that hinders the efficiency of the organization”. Mesdaghinia et al. (69) used the shortened version to measure prohibitive voice (122).

##### 3.3.2.3. Employee voice/speaking-up self-reported behavior

Two distinct measures of self-reported past employee voice behavior were identified across two of the 76 studies included in this review. The Upward Voice Behavior scale by Liu et al. (132) was used in one study (127). The scale consists of three items requiring the participants to indicate the frequency with which they voice their opinions and concerns to their supervisors. The second was an *ad-hoc* questionnaire used by Roussin et al. (70) asking the participants to indicate how often they spoke up in a training program.

##### 3.3.2.4. Speaking-up related climate

The measures in this category include items that aimed at capturing the participants perceptions regarding speaking-up-related climate. Four distinct measures were identified in this category across 16 studies.

Bilotta et al. (128) used an *ad-hoc* scale consisting of three items to measure what they refer to as “organizational-level employee voice”. The three items were related to the perceived organizational climate allowing for voice, initiative and autonomy. Carpini and Flemming (129) reported that they measured speaking up as part of their “Team Behaviors” scale, asking the participants to indicate the extent to which they observed speaking-up behaviors and/or a reluctance to speak up (silence), among other aspects of team behavior. Ridley et al. (77) in their study examining the effectiveness of a teamwork training asked the participants to indicate after each surgical case whether they felt comfortable asking questions and expressing concerns and whether medical errors were reported. Finally, Holland et al. (130) operationalized their approach to voice as that of the “direct voice” defined as occurring in specific two-way communication channels, thus measuring the existence of formal voice channels within the organization.

### 3.4. Theoretical approaches to employee silence and voice

The majority of papers reviewed provide relatively little detail on the theoretical background behind the authors’ approaches to employee voice and silence. Given the paucity of discussion concerning theory construction and development among the papers, simply highlighting these gaps would not be informative or useful. Instead, we have focused on the papers that have attempted to explore theoretical aspects in detail.

Bilotta et al. (128) linked a theory of organizational justice known as the group engagement model (GEM) (129) to employee voice and psychology safety in healthcare. They found robust support for their theoretical model in the healthcare context and found that employee voice accounted for 39% of the variance in the relationship between organizational-level fairness and patient mortality. From a theoretical perspective, the research identified areas for further investigation concerning psychology safety. For example, they found that providers may witness unsafe behavior by their colleagues or supervisor, which in turn leads employees to develop a shared perception of when it is worthwhile to speak up and rely on their fairness perceptions as an indicator that speaking up will not be met with unjust repercussions or unreasonable sanctions. Hu and Casey (67) analyzed employee voice *via* its theoretical links with social identity theory, organizational identification and psychological safety. They found evidence of a complex interaction between safety motivation, psychological safety and safety voice. Specifically, they found that the relationship between safety motivation and safety voice was only significant when psychological safety was low or at an average level. The authors were unable to adequately explain the complex interaction, but they suggest that organizational identification is worthy of more theoretical consideration given that studies have reported that psychological safety can be very low among individuals working in nursing (133). The aforementioned papers should prompt us to consider the speculation of Roussin et al. (70) that “psychological safety microclimates” can help explain the variation in the results regarding the relationship between employee voice/silence and psychological safety. The idea of ‘microclimates’ fits with the suggestion that healthcare organizations are composed of multiple smaller organizations.

Kaya and Bacaksiz (107) examined the relationships between nurses’ positive psychological capital, and their employee voice and organizational silence behaviors. The paper reviewed in detail the concept and theory of positive psychological capital (PsyCap). PsyCap is in line with what is considered to be Positive Organizational Behavior and has four components: self-efficacy, optimism, hope, and resilience (107). Interestingly, the study found that the nurses with higher education levels had lower PsyCap levels, and that nurses with high PsyCap levels remained less silent for individual reasons, but relational reasons cause them to remain silent more. The authors did not adequately explain their contradictory findings, but it further echoes Hu and Casey’s (67) findings concerning the importance of organizational identification. Kesselheim et al. (55) on United States Pediatric trainees provided no information on the theoretical background to their research. However, their results dovetail with aforementioned implications regarding organizational and professional identity (67, 107). Their research on voice among United States Pediatric trainees at 2 large US academic children’s hospitals indicated that, while more than half of the respondents reported observing unprofessional behavior during their most recent inpatient month, strikingly few (20%) respondents anticipated speaking up to an attending in the case of unprofessional behavior, even when they perceived a high risk of patient harm. The observed deficits in speaking up were even more notable considering the vast majority of participants reported prior training specifically related to speaking up. The authors recommend that such stark results call for more assertive communication skills training and anonymous reporting procedures. However, this paper highlights the dangers inherent when there is a lack of any substantive consideration of the drivers (theory) of silence and voice, resulting in recommendations that are generic and cosmetic that cannot be linked back to a suitable evidence base. Research that has closer links between theory, methods and outcomes provides more nuanced outputs. For example, Krenz et al. (93) examined team composition and interprofessional teamwork among healthcare providers and found that stronger hierarchy and more centralized leadership delayed nurses’ voice but did not affect the overall frequency of voice. The researchers directly linked their theory (i.e., contrasting how individuals think they would act and how they actually act) with their method of investigation (i.e., simulation scenarios). Additionally, Loewenbruck et al. (51) studied voice expression among physicians in Germany, Japan and the United States. Their research reviewed the theoretical links between intrinsic and prosocial employee motivation, role-model-based learning power distance and the Theory of Planned Behavior. Contrary to their expectations, internal attribution of medical error prevented voice. The paper is an excellent example of why extensive discussion of theory leads to clearer testable hypotheses that can produce unexpected results, and that contributes to our theorizing about employee voice/silence. Mesdaghinia et al. (69), in a sample of hospital employees working in clinical and administrative positions, connected prohibitive voice with leader-member exchange, moral identity, and moral symbolism. The authors provided an extensive discussion of the theory of moral identity, and specifically assessed moral identity internalization and symbolization. Both these concepts link with the common themes throughout the papers of professional identity and the organizational environment (i.e., organizational identity, psychological safety, organizational level fairness). The reviewed papers generally mentioned classic theories in organizational behavior and occupational health (e.g., Conservation of Resources model, the Job-Demands-Resources model, leadership member exchange theories, social exchange theories and social identity theories) without detailed explanation of how their research links with existing theoretical models and approaches to employee silence/voice. Only one paper approached employee silence/voice directly from an industrial relations perspective. Avgar et al. (49) provide a detailed review of the literature in Labor-Management Partnership (LMP) initiatives, which include direct and indirect methods for eliciting employee input, involvement, and voice. Their research sought to understand why industrial relations approaches have tended to have limited impact on voice. In their research, patient-care voice was enhanced by the positive relationship between quality of LMP processes and employee trust, but the effects of partnership were a function of more than merely participating. In both ANOVA and regression analyses they did not find a significant relationship between participating in an LMP initiative and any of the other variables in the study. Thus, we are left with the unanswered problem of why union effectiveness was not important.

## 4. Discussion

### 4.1. Measures of employee voice/silence

Overall, the 76 studies included in the current IR indicated a high level of heterogeneity regarding the measures of employee voice and silence in healthcare as well as the conceptual descriptions of voice/silence in healthcare. We identified 45 distinct measures, the majority of which was safety-specific and related to employee voice/speaking up. One reason for this heterogeneity could be that different definitions of the concepts are used. This lack of theoretical grounding is directly linked to challenges related to the validity of measures. For one, different theoretical approaches to voice/silence generate different operationalizations, which in turn lead to collecting heterogenous data while claiming to measure the same variable. For example, the approaches of Pinder and Harlos (11) and Tangirala and Ramanujam (12) agree on the intentional withholding of information, but they identify different “targets” from whom employees withhold information; while Pinder and Harlos (11) refer to “persons who are perceived to be capable of effecting change or redress” (p. 334), Tangirala and Ramanujam (12) refer to other members of the workgroup. Such a differentiation, however, has significant implications, as the second case applies to silence between members of the team regardless of their position while the first definition implies withholding information from persons higher in the hierarchy or with power to effect change. Thus, based on the definition, one runs the risk of excluding certain behaviors from the “silence spectrum”; the definition we use to measure and “diagnose” employee silence will shape the findings and these in turn, will shape interventions and policies. Congruently, voicing concerns can often take the form of venting or complaining about a colleague’s or superior’s behavior; formulating “behavioral prototypes” on what is desirable and what not in terms of voice behaviors could potentially discourage genuine expression and sharing of crucial information—like for example in a strong culture of conflict avoidance (134). Moreover, very few studies collected data on the positive forms of employee silence (e.g., prosocial silence), which runs the risk of suggesting that there are no positive aspects to silence in the workplace. Thus, there is a research gap regarding the positive reasons for employee silence. Congruently, there is a significant heterogeneity in the measures identified and there seems to be a narrow understanding of what are the “appropriate” ways of speaking up, which might result in excluding important ways of sharing information.

A significant amount of research only investigated speaking-up content related to patient safety and quality of care—with some measures being developed on the overlap of employee voice with stating safety concerns (88) or with error disclosure (51). This runs the risk of suggesting an overlap between speaking-up for patient safety and employee voice in healthcare. While speaking-up for patient safety is of crucial importance, it remains unclear what other forms and content might be relevant to the employee voice notion in healthcare, and whether other important issues not directly related to patient safety concerns are being hindered when the focus is solely on patient safety and/or error reporting. Congruently, the studies involving external observation of speaking-up behaviors were directly related to patient safety concerns, and almost all occurred within simulation or training settings and in a rather controlled environment. In terms of ecological validity, it is impossible to know whether the findings could be replicated in real work settings, due to the numerous limitations associated with observational studies employing trainings and/or simulations.

Similarly, our review has identified several measures of employee voice/silence and speaking up that consist of items related to the participants’ perceptions of whether speaking-up is valued or frowned upon in their team/organization; such measures provide us with important insights into the voice-related culture of healthcare teams and organizations. However, it is rather unclear how these measures are related to or differentiated from similar measures of organizational culture and work-related climate (6, 135), as well as the potential conceptual/theoretical overlap with other concepts like psychological safety and teamwork climate.

Differences in interpretation will lead to different ways of formulating and addressing the problem. This will become an even bigger problem when the justification of measuring voice or silence in a certain way is not clearly related to a specific definition. The fragmentation related to the silence and voice research does however not only emerge from differences in concept definitions, but also results from an unstructured framework related to the two concepts. The various antecedents (e.g., condition and situations, issues that trigger voice/silence response, motives) are all mixed in various ways. Motives for silence are for example sometimes understood as feelings or conditions and other times as situations, while also being classified as forms of silence and/or voice. As indicated by the studies that use the Van Dyne et al. (53) scale, the types of prosocial silence, acquiescent silence and defensive silence emerge from associated motives, while Yalcin and Baykal (40) measured silence motives as silence climate, silence based on fear, acquiescent silence and silence based on protecting the organization. Thus, motives can be both feelings (e.g., fear) and can indicate perceptions of organizational conditions as well as the type of context which triggers the silence behavior (e.g., a relational context in which one wants to protect the organization or to cooperate smoothly with colleagues). Overall, the concepts of voice and silence are understood in very heterogenous ways, and the ways in which voice and silence are framed are fragmented and bleary. This creates a vague starting point when trying to identify suitable interventions in order to create conditions that promote voice in healthcare organizations and might be a reason for why existing interventions seem to fail.

The vast majority of the reviewed research relies upon self-reported measures—with the exception of the seven studies involving external observation. This means that any attempt to map the silence/voice distinction—meaning whether what is observed is behavioral activation or behavioral inhibition (13)—is highly dependent on the employees’ subjective experiences and perceptions of whether and when engaging in voice behaviors is viewed positively or negatively by their team, supervisors and/or organization or their profession. Research in the field of social media usage has indicated significant discrepancies between self-reported data on time spent on social media versus objective data collected *via* monitoring the users’ devices (136). In simple terms, we cannot claim that what we know about employee silence and voice in healthcare *via* self-reported measures is what is really happening, but it is rather what employees remember, perceive, think and want to report when given the opportunity to participate in a study, as well as their perceptions of how “safe” speaking-up might be for their career and their team. And presumably also, this is limited by what they consider being included in “speaking up”; for example, some employees might think “speaking up” only means making an official report, or conversely, that reporting does not constitute speaking-up, but only verbal mentions do.

This brings us back to the importance of the context, the persons involved and the content of silence/voice. As Okuyama et al. (137) point out, a healthcare employee might consider differently the pros and cons of speaking up if they are about to share an innovative idea in a brainstorming session or a concern about patient safety in the operating room. Noort et al. (138) in their review of safety voice in healthcare, highlight the uniqueness of safety voice. Thus, the content and the context of speaking-up can differentiate the extent to which silence or voice can be considered discretionary, as noted in the recent paper by Creese et al. (139), which highlights a distinction healthcare staff draw between speaking up for patient wellbeing and speaking up for their own wellbeing. Concealing a medical error, for instance, implies ethical responsibility, moral injury and legal ramifications—and withholding safety voice can be considered illegal (138). On the other hand, keeping one’s innovative idea to oneself during a general discussion is less (directly) damaging. Thus, it is not clear yet to what extent healthcare staff are engaging into conscious processing and evaluations in different situations when they decide whether to speak-up or withhold voice; whether this changes over the career span or across the hierarchy; or whether individual intention to speak up is mostly overpowered by external influencing factors and potential consequences that exceed any bearable costs—leading to whistleblowing being the solution when a disaster point has been reached (140, 141). Our review showed an increased interest in studying healthcare employees’ perceptions of how safe it is for them to speak-up when patient safety is at stake—but very little focus on systemic and contextual factors (e.g., leader-member exchange). Such focus on the potential systemic failures of healthcare organizations suggests that the study of employee silence/voice in healthcare would benefit more from the inclusion of an Industrial Relations approach to voice, which can potentially help better understand the organizational and professional contexts, institutional antecedents and mechanisms for employee voice and silence (142).

As far as reasons for employee silence and/or motives for speaking up are concerned, similar limitations can apply here as well; the different classifications developed in the literature for silence and voice based on potential motives (e.g., acquiescent silence, prosocial silence, etc., or prosocial voice, defensive voice, etc.) have led to measures that ask participants to indicate “How often have you remained silent because you are afraid of retaliation?”. There is no way to tell whether participants’ answers to those questions have been affected by either a recent negative experience where sharing one’s concerns lead to significant negative consequences or an internalized belief that in healthcare silence might lead to better career opportunities of speaking up against superioris is not welcome. The propensity of organizational research to translate subjective data, that is susceptible to several biases, into “objective” observations about the team, organization, system or industry, has been heavily criticized after recent meta-analyses indicated that organizational research runs the risk of becoming irrelevant, since only 1.5% of research actually incorporates implications for practice and policy (143), and the vast majority of research remains theoretical and severely limited by methodological artifacts and biases.

### 4.2. Theoretical implications

All authors cited relevant research on the topic and recognized the relationship between the sharing of important information and its impact on healthcare delivery. However, there was a general lack of theoretical consideration, and even the papers which discussed relevant theory failed to explicitly link their study variables to a direct examination of the named theories. The lack of success in the area of theory construction in psychology (and the social sciences generally) and the aversion to addressing this problem has been reviewed in detail (144–148). Most of the studies included in this review implied that healthcare professionals can be overpowered by external influencing factors in terms of their willingness to speak-up. This is consistent with observations that the power imbalance between employees and organizations happens in parallel with the low levels of control over healthcare employees’ day-to-day work life, as has been highlighted in the job burnout literature with the Job Demands-Control Model (JDC) (21, 22) and the Job Demands-Resources Model (JDR) (22, 23). Thus, there is a need for a more rigorous and robust approach to the links between research and theoretical background both in terms of employee silence/voice as theoretical constructs as well as regarding the relationships between the voice/silence and other variables/concepts. A good example of an attempt to link employee silence with employee outcomes is the study conducted by Knoll et al. (19), which included a longitudinal examination of the relationship between employee silence motives and burnout. The paper provides empirical and theoretical justification for measuring employee silence (as opposed to employee voice), is consistent in focusing on employee silence motives and provides explanatory links with the variable of interest (burnout).

In terms of employee voice/silence as theoretical constructs, acknowledging the theoretical and operational definitions in which the different measures of employee voice/silence are rooted is of critical importance. The current review suggests that the measures used in the reviewed studies reflect a variety of operationalizations (e.g., intentions or self-reported past behaviors or motives, self *VS* organizational climate) and approaches (e.g., Organizational Behavior *VS* Industrial Relations approach) and variations in the content (e.g., safety voice). Thus, there is a need for better justification of the chosen measure for employee voice/silence which should also be reflected in the rationale of each study.

In terms of the relationships between voice/silence and other variables explored in the reviewed studies, there is a need for clearer explanatory frameworks with a more comprehensive understanding of how voice/silence is approached and thus linked with other constructs. For example, measuring employee voice/silence motives has different theoretical implications compared to assessing speaking up within observational rubrics in simulation settings. Additionally, it remains unclear when and why researchers choose to measure employee voice or employee silence, with employee silence often being discussed as an indicator of “absence of voice”. Given that two meta-analyses across different industries have suggested that voice and silence are related to different antecedents and are differently related to consequences (13, 149), it is important to draw on empirical evidence and theoretical background in order to justify whether employee voice or silence (or both) should be measured in relationship to other variables of interest. The reviewed literature fails to reflect the complexity surrounding employees’ withholding or sharing of information in healthcare, in that (a) silence and voice are not mutually exclusive, meaning that healthcare staff might voice certain concerns but withhold others; (b) voice/silence can occur on different levels, meaning that speaking up to team members might be easier compared to speaking up to superiors in the organization; and (c) any self-reported assessment of employee silence/voice carries significant information about the individuals’ experiences of the organizational culture, individual predispositions as well as aspects related to their professional identity and “lessons” from their career trajectory since medical education.

Overall, the lack of substantive discussion of theory represents a barrier to progress in the field. According to Borsboom et al. (144), a lack of explanatory theories can hinder progress in a field in three ways. First, it creates the danger of ‘inventing the wheel’ over and over again because we do not understand how different phenomena relate to each other. Second, without strong theories we cannot identify the most effective interventions for changing a system in the desired way. Third, without comprehensive theories we often do not know where to look when designing new studies.

### 4.3. Implications for practice

Communication within healthcare organizations cannot be limited to promoting voice channels that only allow suggestions for improvement aligned with the organizational goals—especially when these goals are in times conflicting (e.g., patient safety goals can often be in conflict with the financial goals of the health care organization). The importance of developing a voice/silence framework that incorporates macro-level factors is highlighted by research findings indicating that “normalization” of employee silence can potentially be a side-effect of a shallow voice system, whereby the existence of voice channels is merely a front for a rather non-receptive leadership (142). Research evidence suggests that several macro-level and institutional factors (such as Market Economies; political characteristics; institutional employment regime) can have a definitive impact on employee voice and silence (150). An approach to voice and silence that integrates Industrial Relations with HRM/OB will allow a richer explanation of when, why and how employees decide to speak up or to remain silent, incorporating the power dynamics and conflicts of interest between employers and employees, as well as the power imbalances rendering from the hierarchy, especially in healthcare, where hierarchy is strict within the team (e.g., nurses versus physicians; attending physicians versus residents; physicians versus board members, etc.). To put it simply, understanding why, when and how healthcare employees choose to speak up or to remain silent—and identifying what should be considered as voice—cannot be done without taking into account organizational and industrial macro-level factors.

We mentioned earlier that a significant proportion of the papers in this review mainly addressed voice and silence from a patient safety perspective (safety voice). This is, evidently, a very important aspect of healthcare. However, moving forwards, it is also important to understand the concepts of voice and silence from an organizational improvement perspective as well as from an employee perspective, especially as voice and silence can vary depending on the type of situation [e.g., (67, 139)]. To be able to delineate practical implications, more research is required to determine how different types of situations might trigger different patterns of voice and silence among healthcare professionals. Patient safety issues might trigger certain types of patterns while organizational development issues might trigger others. Very few studies focused mainly on the organizational improvement perspective while most addressed the employees’ perspective and self-reported behavior.

On the individual level, factors such as motives (127), values (104), competence (124) and self-efficacy (70) were found to be associated with voice or silence. The individual perspective on voice and silence will of course continue to be important, as teams and organizations consist of individuals, but a far less researched perspective is that of silence at the team level. Team communication has been consistently identified as a critical component of effective teams and efficient organizations. The literature on what can make a team more effective is constantly growing, aiming to identify those aspects of team performance that can explain why some teams are better than others—and that has also included a focus not only on *what* is being communicated, but also how (151, 152). While some aspects of effective teamwork might be relevant to most industries and sectors, no empirical study or theoretical model can claim that the one-size-that-fits-all has been found. In the studies that also looked at the team level, team-based self-esteem, and trust (118), status of team members (94) and horizontal violence among peers (38) were identified as significant factors. Also, evidence that suggests that groups belonging to welfare-professions such as nurses voice their concerns less often than groups belonging to classical professions, such as doctors and psychologists (38, 40, 98) can have important implications for practice and policy, indicating that staff lower in the hierarchy need more supportive policies to be able to speak up equally to staff higher in the hierarchy of the team. One important aspect of being able to address future challenges will be to transcend traditional borders and cooperate both within and across professional groups, levels of care and organizations. Multi-disciplinary groups will, moreover, grow in importance as digitization becomes further embedded into healthcare and new professional groups, such as IT-specialists and business developers, join healthcare teams.

Looking at the level of leadership, factors such as leadership styles (78, 106, 110, 119, 126, 127), supervisor’s job insecurity (66) and leadership centralization (93) were among those associated with voice and silence among employees. Although 16 papers directly addressed the role of leadership related to employee voice or silence, the voice or silence of managers themselves has been sparsely analyzed. First-line managers can be very important agents in bridging the relationship between professionals and the organization going forward. It is important that first-line/mid-level managers voice concerns both up and down the hierarchy, including issues beyond patient safety, such as career follow-up processes, compensation structures, budget and administrative practices that need to be addressed for the organization to be able to adapt to external changes. On the organizational level, factors such as workplace toxicity (113), organizational-level fairness (128), labor–management partnership (49), organizational culture (81) as well as type of role, discipline and hospital (55, 98) were found to significantly affect voice and silence behaviors.

Although 22 of the studies included in this review referred to how different organizational factors could affect voice behaviors, there was very limited focus on how these factors actually affect employees’ willingness to engage in organizational development issues. One important organizational factor that was not analyzed in the reviewed studies, but that is central to both patient safety, organizational development and employee turnover, is organizational control. For example, answering questions like how different types of control systems help or hinder employees to voice concerns related to organizational development and employee issues and whether different forms of silence are related to different types of control systems can significantly contribute to our understanding of how to address employee silence in healthcare.

**Figure 2:**
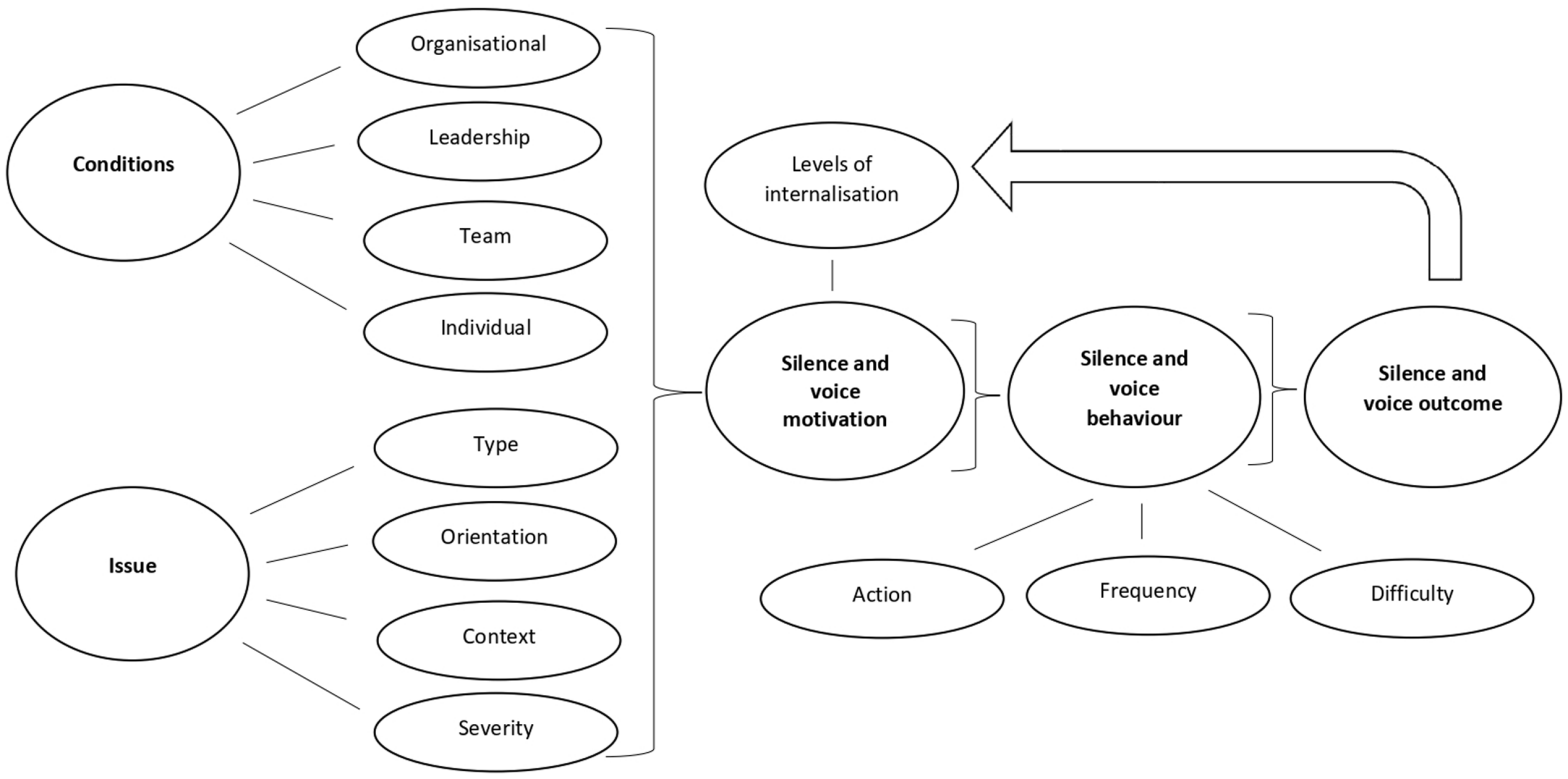
Framework of conditions and issues affecting employee voice and silence.

Given the current landscape of research related to voice and silence, we suggest that a more transparent and clearer framework should be used that could encompass both silence and voice. We have included an example of a potential framework (see Figure 2) which indicates that aspects of motivation should be clearly separated from the conditions, type of issues, issue-orientation, context, issue severity, behaviors and outcomes of silence. Such a framework can help researchers and practitioners address different aspects of voice and silence in a more structured way, and also improve the capability of identifying suitable interventions in healthcare organizations. At the center of this framework lies motivation for voice and silence, which of course then means that the understanding of motivation in relation to silence and voice needs to be elucidated. Motivation theory could lead to an interesting way to evaluate voice and silence and be understood in terms of different levels of internalization (152) of voice and silence behavior, that could be a fruitful way forward.

### 4.4. Limitations

Regarding the studies included in this review, the majority of the studies employed self-reported measures of silence or/and voice, thus collecting data on employees’ perceptions of how often they do speak up/remain silence due to specific reasons (motives for silence/voice); regarding specific content (focused on patient safety or other issues) or employees’ perception of issues related to speaking up/silence climate in their team or their organization. We discussed the limitations related to the conceptualization and measurement in detail previously, however, it is necessary to emphasize that each source of information can provide us with important insights regarding employees’ perceptions of silence and/voice in their team or their organizations, as well as the self-reported voice behaviors, as they indicate how they experience their work environment with regard to voice/silence. The studies were significantly heterogeneous in terms of measures used, sample sizes, theoretical background and examined outcomes. Most studies were using a cross sectional design, which significantly affected the results of the quality assessment as well as the validity of their findings. Our knowledge of employee voice/silence in healthcare is mainly based on self-report measures with few observations of simulated situations, which could have been enriched by more field research.

It is important to note that more than 50% of the participants belonged to the nursing profession and approximately 70% identified as women; thus, the review findings might be more representative of the research and experiences of nursing professionals and of women working in healthcare. To achieve a better understanding of the complex phenomena of employee silence and voice in healthcare, future research needs to address all involved professional groups; this will also allow the future design of organization-level interventions with a systematic approach and an objective to create healthy workplaces for all employees. The vast majority of the studies did not provide detailed information on the departments/units where the research was carried out; however, in contrast to the frequently reported “WEIRD” problem—meaning that the majority of research is usually carried out in western and developed countries—approximately 50% of the studies included in this IR were conducted in countries outside of Europe, the United States and North America. To that end, future research can incorporate socio-cultural variables such as power orientation and individualism/ collectivism which can affect silence/voice behaviors (153), team communication (118) and professional identity (154).

The current review focused on quantitative studies measuring employee voice and/or silence directly and/or indirectly among healthcare workers and we only included research studies that were published from January 2016 to January 2022; thus, we cannot be certain if our results would be different if we were to include earlier studies as well. Our review aimed at exploring how employee silence/voice have been measured in healthcare, however, future reviews are necessary to assess the quality of measurement in more detail based on theory, scientific foundation and statistical foundation. The current review was also limited by the file-drawer problem (155), whereby it is possible that important unpublished work may have been missed. We are not able to know if/and to what extent there was a selective publication bias and whether there were studies that remained unpublished due to non–statistically significant results. Although we searched eight well–known databases, we still cannot be certain whether all the studies that examined employee silence/voice among healthcare professionals during the selected time period were identified and included in this IR. Additionally, only articles in English were reviewed. The heterogeneous nature and quality of the studies was also a barrier to synthesizing them in a more meaningful way. Additionally, the IR only included quantitative studies, whereas qualitative studies could have provided more detailed information on the mechanisms and factors underlying the phenomena of employee voice and silence in healthcare.

## 5. Conclusion

Overall, the heterogeneous measures of employee silence and voice indicated a lack of consensus regarding what we should measure when conducting research about employee silence and employee voice in healthcare. The key theoretical, research and practical implications are listed in Table 4. The complexities of the healthcare industry require a new framework whereby employee voice is analyzed within the different contexts and situations it may occur, as what drives safety voice is probably considerably different from what drives general employee voice; thus, it is not surprising that interventions to encourage speaking up in healthcare have been reporting disappointing results, since it is still unclear what exactly these interventions aim at achieving and how they can do that.

**Table 4:** Summary of key-points for theoretical, research and practical implications.

Implications for theory
– Lack of overarching or comprehensive theory concerning employee silence and employee voice in healthcare
– Heterogeneous coverage of mechanisms, with no clear agreement on whether the phenomena are better understood as antecedents or outcomes
– Repeated assertions that silence/voice are connected with wellbeing without adequate explanatory models that link job-stress models with silence/voice
Implications for research
– Lack of clarity on what constitutes voice/silence results in fragmented approaches to measurement
– Existing measures are dominated by the proposition that silence is always negative, and voice is always positive
– The lack of longitudinal data and reliance on self-report data mean that our knowledge about voice/silence in healthcare is tenuous and needs further verification
Implications for practice
– Knowing the micro, meso and macro factors that influence why, when and how healthcare employees choose to speak up or to remain silent is important for implementation
– Academic research needs to pay more attention to how and when voice can aid organizational improvement
– One size does not fit all, meaning that different solutions are needed for different contexts

## Author contributions

OL and AM contributed equally to the conceptualization and original ideas of the paper, developed and designed the methodology. AM supervised the intellectual and organizational genesis of the paper. OL, MJ, and AM conducted data collection and synthesized the data. OL and AM prepared the first draft of the paper. MJ contributed to the first draft of the paper. CM, KP, and CC conducted the quality assessment. JJ and EK contributed towards writing, editing, and reviewing subsequent versions of the paper. All authors contributed to the article and approved the submitted version.

## Conflict of interest

The authors declare that the research was conducted in the absence of any commercial or financial relationships that could be construed as a potential conflict of interest.

## Publisher’s note

All claims expressed in this article are solely those of the authors and do not necessarily represent those of their affiliated organizations, or those of the publisher, the editors and the reviewers. Any product that may be evaluated in this article, or claim that may be made by its manufacturer, is not guaranteed or endorsed by the publisher.
