# Supplementary material for: An integrative systematic review of employee silence and voice in healthcare: what are we really measuring?
Source: Front Psychiatry. 2023 May 25;14:1111579. doi: 10.3389/fpsyt.2023.1111579 (PMC10248453; doi:10.3389/fpsyt.2023.1111579)
Supplement: Supplementary file 1 [file Data_Sheet_1.PDF]

Supplemenytary Material. Search Strings for multiple databases

**PubMed**
("Employee silenc*"[Title/Abstract] OR "Employee voic*"[Title/Abstract] OR "Organizational silenc*"[Title/Abstract] OR "Organisational silenc*"[Title/Abstract] OR "Organizational voic*"[Title/Abstract] OR "Organisational voic*"[Title/Abstract] OR "Silence behaviour"[Title/Abstract] OR "Voice behaviour"[Title/Abstract] OR "Voice behavior"[Title/Abstract] OR "Speak* up"[Title/Abstract] OR "Prohibitive voic*"[Title/Abstract] OR "Promotive voic*"[Title/Abstract]) OR "speak-up related climate"[Title/Abstract] OR "silence culture"[Title/Abstract] OR Concealment[Title/Abstract] OR "Truth disclosure"[Title/Abstract] OR "Transparency"[Title/Abstract] OR "Error concealment"[Title/Abstract] OR Confidentiality[Title/Abstract] OR "medical error*"[Title/Abstract] OR "rais* concerns"[Title/Abstract] OR Whistleblow*[Title/Abstract] OR Whistle-blow*[Title/Abstract] OR "blowing the whistle"[Title/Abstract])
AND
("Healthcare"OR "health organization"OR "health organisation" OR "health service"OR "medical service" OR hospital*OR "primary care" OR "health personnel" OR "health personnel" OR "health employee" OR "healthcare employee" OR nurs* OR physician* OR doctor* OR medic* OR “patient safety”)
**Scopus**
TITLE-ABS-KEY (("Employee silenc*" OR "Employee voic*" OR "Organizational silenc*" OR "Organisational silenc*" OR "Organizational voic*" OR "Organisational voic*" OR "Silence behaviour" OR "Voice behaviour" OR "Voice behavior" OR "Speak up" OR "Prohibitive voic*" OR "Promotive voic*" OR "speak-up related climate" OR "silence culture" OR "Concealment" OR "Truth disclosure" OR "Transparency" OR "Error concealment" OR "Confidentiality" OR "medical error*" OR "rais* concerns" OR "Whistleblow*" OR "Whistle-blow*" OR "blowing the whistle"))
AND
ALL (("Healthcare" OR "health organization" OR "health organisation" OR "health service" OR "medical service" OR "hospital*" OR "primary care" OR "health personnel" OR "health personnel" OR "health employee" OR "healthcare employee" OR "nurs*" OR "physician*" OR "doctor*" OR "medic*"))

**PsychInfo**
noft("Employee silenc*" OR "Employee voic*" OR "Organizational silenc*" OR "Organisational silenc*" OR "Organizational voic*" OR "Organisational voic*" OR "Silence behaviour" OR "Voice behaviour" OR "Voice behavior" OR "Speak up" OR "Prohibitive voic*" OR "Promotive voic*" OR "speak-up related climate" OR "silence culture" OR Concealment OR "Truth disclosure" OR "Transparency" OR "Error concealment" OR Confidentiality OR "medical error*" OR "rais* concerns" OR Whistleblow* OR Whistle-blow* OR "blowing the whistle")
AND
("Healthcare" OR "health organization" OR "health organisation" OR "health service" OR "medical service" OR hospital* OR "primary care" OR "health personnel" OR "health personnel" OR "health employee" OR "healthcare employee" OR nurs* OR physician* OR doctor* OR medic*)
**Google Scholar**
intitle:"Employee silence" OR "Employee voice" OR "Organizational silence" OR "Organisational silence" OR "Organizational voice" OR "Organisational voice"
intitle "Silence behaviour" OR "Voice behaviour" OR "Voice behavior" OR "Speak up" OR "Prohibitive voice" OR "Promotive voice" OR "speak-up related climate" OR "silence culture"
intitle:Concealment OR "Truth disclosure" OR Transparency OR "Error concealment" OR Confidentiality OR "medical error" OR "raise concerns" OR Whistleblow OR “blowing the whistle"
AND
(Healthcare OR "health organization" OR "health organisation")
("health service" OR "medical service" OR hospital* OR “patient safety”)
("primary care" OR "health personnel" OR "health personnel")
("health employee" OR "healthcare employee" OR nurse)
(physician OR doctor OR medic*)

**CINAHL**
((TI "Employee silenc*" OR AB "Employee silenc*") OR (TI "Employee voic*" OR AB "Employee voic*") OR (TI "Organizational silenc*" OR AB "Organizational silenc*") OR (TI "Organisational silenc*" OR AB "Organisational silenc*") OR (TI "Organizational voic*" OR AB "Organizational voic*") OR (TI "Organisational voic*" OR AB "Organisational voic*") OR (TI "Silence behaviour" OR AB "Silence behaviour") OR (TI "Voice behaviour" OR AB "Voice behaviour") OR (TI "Voice behavior" OR AB "Voice behavior") OR (TI "Speak* up" OR AB "Speak* up") OR (TI "Prohibitive voic*" OR AB "Prohibitive voic*") OR (TI "Promotive voic*" OR AB "Promotive voic*") OR (TI "speak-up related climate" OR AB "speak-up related climate") OR (TI "silence culture" OR AB "silence culture") OR (TI Concealment OR AB Concealment) OR (TI "Truth disclosure" OR AB "Truth disclosure") OR (TI Transparency OR AB Transparency) OR (TI "Error concealment" OR AB "Error concealment") OR (TI Confidentiality OR AB Confidentiality) OR (TI "medical error*" OR AB "medical error*") OR (TI "rais* concerns" OR AB "rais* concerns") OR (TI Whistleblow* OR AB Whistleblow*) OR (TI Whistle-blow* OR AB Whistle-blow*) OR (TI "blowing the whistle" OR AB "blowing the whistle"))
AND
(Healthcare OR "health organization" OR "health organisation" OR "health service" OR "medical service" OR hospital* OR "primary care" OR "health personnel" OR "health personnel" OR "health employee" OR "healthcare employee" OR nurs* OR physician* OR doctor* OR medic* OR “patient safety”)
**Embase (via OVID)**
("Employee silenc*":ti,ab OR "Employee voic*":ti,ab OR "Organizational silenc*":ti,ab OR "Organisational silenc*":ti,ab OR "Organizational voic*":ti,ab OR "Organisational voic*":ti,ab OR "Silence behaviour":ti,ab OR "Voice behaviour":ti,ab OR "Voice behavior":ti,ab OR "Speak* up":ti,ab OR "Prohibitive voic*":ti,ab OR "Promotive voic*":ti,ab OR "speak-up related climate":ti,ab OR "silence culture":ti,ab OR Concealment:ti,ab OR "Truth disclosure":ti,ab OR Transparency:ti,ab OR "Error concealment":ti,ab OR Confidentiality:ti,ab OR "medical error*":ti,ab OR "rais* concerns":ti,ab OR Whistleblow*:ti,ab OR Whistle-blow*:ti,ab OR "blowing the whistle":ti,ab)
and

(Healthcare OR "health organization" OR "health organisation" OR "health service" OR "medical service" OR hospital* OR "primary care" OR "health personnel" OR "health personnel" OR "health employee" OR "healthcare employee" OR nurs* OR physician* OR doctor* OR medic* OR “patient safety”)
**Web of Science**
(AB=("Employee silenc*" OR "Employee voic*" OR "Organizational silenc*" OR "Organisational silenc*" OR "Organizational voic*" OR "Organisational voic*" OR "Silence behaviour" OR "Voice behaviour" OR "Voice behavior" OR "Speak* up" OR "Prohibitive voic*" OR "Promotive voic*" OR "speak-up related climate" OR "silence culture" OR Concealment OR "Truth disclosure" OR Transparency OR "Error concealment" OR Confidentiality OR "medical error*" OR "rais* concerns" OR Whistleblow* OR Whistle-blow* OR "blowing the whistle”)
AND
ALL FIELDS: ((Healthcare OR "health organization" OR "health organisation" OR "health service" OR "medical service" OR hospital* OR "primary care" OR "health personnel" OR "health personnel" OR "health employee" OR "healthcare employee" OR nurs* OR physician* OR doctor* OR medic* OR “patient safety”))
**Cochrane**
(("Employee" NEAR/2 silenc*):ti,ab OR ("Employee" NEAR/2 voic*):ti,ab OR ("Organizational" NEAR/2 silenc*):ti,ab OR ("Organisational" NEAR/2 silenc*):ti,ab OR ("Organizational" NEAR/2 voic*):ti,ab OR ("Organisational" NEAR/2 voic*):ti,ab OR "Silence behaviour":ti,ab OR "Voice behaviour":ti,ab OR "Voice behavior":ti,ab OR (Speak* NEAR/2 "up"):ti,ab OR ("Prohibitive" NEAR/2 voic*):ti,ab OR ("Promotive" NEAR/2 voic*):ti,ab OR "speak-up related climate":ti,ab OR "silence culture":ti,ab OR Concealment:ti,ab OR "Truth disclosure":ti,ab OR Transparency:ti,ab OR "Error concealment":ti,ab OR Confidentiality:ti,ab OR ("medical" NEAR/2 error*):ti,ab OR (rais* NEAR/2 "concerns"):ti,ab OR Whistleblow*:ti,ab OR Whistle-blow*:ti,ab OR "blowing the whistle":ti,ab)
AND
(Healthcare OR "health organization" OR "health organisation" OR "health service" OR "medical service" OR hospital* OR "primary care" OR "health personnel" OR "health personnel" OR "health employee" OR "healthcare employee" OR nurs* OR physician* OR doctor* OR medic* OR “patient safety”)
