# Supplementary material for: An integrative systematic review of employee silence and voice in healthcare: what are we really measuring?
Source: Front Psychiatry. 2023 May 25;14:1111579. doi: 10.3389/fpsyt.2023.1111579 (PMC10248453; doi:10.3389/fpsyt.2023.1111579)
Supplement: Supplementary file 2 [file Data_Sheet_2.PDF]

No.	Article (Alphabetical)	1	2	3	4	5	6	7	8	9	10	11	12	13	14	Score
1.	Abd El-Fattah Mohamed Aly et al., (2021)	Y	N	Y	N	N	N	N/A	Y	Y	N	N	N/A	N/A	N	4/14
2.	Abdelmotale et al., (2021)	Y	N	Y	N	N	N	N/A	Y	Y	N	Y	N/A	N/A	Y	6/14
3.	Abdullah Mohamed et al., (2021)	Y	Y	N/R	Y	N	N	N/A	Y	Y	N	Y	N/A	N/A	N	6/14
4.	Al-Abrrow (2018)	Y	Y	N	N	N	N	N/A	Y	Y	N	Y	N/A	N/A	N	5/14
5.	Alheet (2019)	Y	Y	N	Y	N	N	N/A	Y	N	N	Y	N/A	N/A	N	5/14
6.	Alingh et al., (2018)	Y	Y	N	Y	N	N	N/A	Y	Y	N	Y	N/A	N/A	Y	7/14
7.	Amar et al. (2019)	Y	Y	N	N	N	N	N/A	Y	Y	N	N	N/A	N/A	N	4/14
8.	Amiri et al., (2018)	Y	Y	N	Y	N	N/A	N	Y	Y	N	Y	CD	Y	N	7/14
9.	Aslan et al. (2021)	Y	Y	Y	Y	Y	N	N/A	Y	Y	N	Y	N	N/A	Y	8/14
10.	Avgar et al. (2016)	Y	Y	N	Y	N	N	N/A	Y	Y	N	Y	N	N/A	Y	7/14
11.	Best & Kim (2019)	Y	Y	N	N	N	N	N/A	Y	Y	N	N	CD	N/A	N	4/14
12.	Bilotta et al. (2021)	Y	Y	Y	CD	Y	N	N/A	Y	Y	N	Y	N/A	N/A	Y	8/14
13.	Carpini & Flemming (2020)	Y	Y	N	CD	Y	N	N/A	Y	N	N	N	N/A	N/A	N	4/14

14.	Çaylak & Altuntas, (2017)	Y	Y	Y	Y	N	N	N/A	Y	Y	N	Y	N/A	N/A	N	7/14
15.	Chang et al. (2019)	Y	Y	Y	CD	N	N	N/A	Y	Y	N	Y	N/A	N/A	N	6/14
16.	D'Agostino et al. (2017)	Y	Y	CD	Y	CD	N	N/A	Y	Y	N	Y	C/D	N/A	N	6/14
17.	Darawad et al. (2020)	Y	Y	Y	Y	N	N	N/A	Y	Y	N	Y	N/A	N/A	N	7/14
18.	De los Santos et al. (2020)	Y	Y	Y	Y	N	N	N/A	Y	Y	N	Y	N/A	N/A	Y	8/14
19.	Doo & Choi, (2020)	Y	Y	C/D	Y	Y	N	N/A	Y	Y	N	Y	N/A	N/A	C/D	7/14
20.	Doo & Kim, (2020)	Y	Y	Y	Y	Y	N	N/A	Y	Y	N	Y	N/A	N/A	N/R	8/14
21.	Erkutlu & Chafra (2018)	Y	Y	Y	Y	Y	N	N/A	Y	Y	N	Y	N/A	N/A	Y	9/14
22.	Gauld et al. (2020)	Y	Y	N	Y	Y	N	N/A	Y	Y	C/D	Y	N/A	N/A	Y	8/14
23.	Ginsburg & Bain (2017)	Y	Y	Y	Y	Y	Y	Y	Y	Y	Y	Y	C/D	N	N	11/14
24.	Gong et al., 2021	Y	Y	Y	Y	Y	Y	Y	Y	Y	N	Y	N/A	N/A	Y	11/14
25.	Gkorezis et al., (2016)	Y	Y	Y	Y	Y	N	N/A	N/A	Y	N	Y	N/A	N/A	Y	8/14
26.	Guo et al., 2021	Y	Y	N	Y	Y	N	N/A	N/A	Y	N	Y	N/A	N/A	Y	8/14
27.	Gupta & Ravindranath (2016)	Y	Y	N	Y	Y	N	N/A	N/A	Y	N	Y	N/A	N/A	Y	8/14
28.	Guris et al., (2019)	Y	Y	Y	Y	Y	Y	Y	Y	Y	Y	Y	Y	Y	Y	14/14

29.	Henkin et al. (2016)	Y	Y	Y	Y	N	Y	Y	Y	Y	N	Y	N	N	N	9/14
30.	Herrington & Hand, (2018)	Y	Y	N/R	Y	Y	Y	Y	N	Y	N	Y	N/A	N/A	Y	9/14
31.	Holland et al., 2017	Y	Y	N	Y	Y	Y	N/A	N/A	Y	N	Y	N/A	N/A	Y	8/14
32.	Hu & Casey (2021)	Y	Y	N	Y	Y	N	N/A	N/A	Y	N	Y	N/A	N/A	NR	6/14
33.	Islam et al., (2017)	Y	Y	Y	Y	Y	N	N/A	N/A	Y	N	C/D	N/A	N/A	N/R	6/14
34.	Jeong et al., (2021)	Y	Y	Y	Y	Y	N	N/A	Y	Y	N	Y	N/A	N/A	Y	9/14
35.	Jungbauer et al. (2018)	Y	Y	N	Y	N	N	C/D	N/A	Y	N	Y	N/A	N/A	N	5/14
36.	Kaya & Eskin Bacaksiz (2021)	Y	Y	N	Y	N	N	N	Y	Y	N	Y	N/A	NA	Y	7/14
37.	Kesselheim et al., (2021)	Y	Y	N	Y	Y	N	N	Y	Y	N	NR	NA	NA	NR	6/14
38.	Krenz et al., (2020)	Y	Y	C/D	Y	N	N	N	Y	N/A	N	Y	CD	NA	N	5/14
39.	Kritsotakis et al., (2021)	Y	Y	Y	Y	N	N	N	Y	Y	N	Y	N/A	NA	N	7/14
40.	Labrague & De los Santos (2020)	Y	Y	Y	Y	N	N	N	Y	NA	N	Y	N/A	NA	N	6/14
41.	Lawson et al. (2017)	Y	Y	CD	Y	N	N	N	Y	Y	N	Y	N/A	NA	CD	6/14
42.	Lee & Dahinten (2021)	Y	Y	Y	Y	Y	N	N	Y	Y	N	Y	N/A	NA	N	8/14
43.	Lemke et al. (2021)	Y	Y	CD	Y	N	N	N/A	Y	Y	N	Y	CD	NA	N	6/14

44.	Loewenbruck et al., (2016)	Y	Y	CD	Y	N	N	N	Y	N	N	N	Y	NA	Y	6/14
45.	Luff et al., (2021)	Y	Y	CD	Y	NR	Y	CD	Y	Y	N	Y	CD	Y	N	8/14
46.	MacMahon et al., (2018)	Y	Y	N	Y	N	N	N	Y	Y	N	Y	CD	NA	N	6/14
47.	Manapragada & Bruk-Lee, (2016)	Y	Y	CD	Y	N	N	N	Y	Y	N	Y	Y	NA	Y	8/14
48.	Mansour et al., (2020)	Y	Y	Y	Y	N	N	N	Y	Y	N	Y	CD	NA	N	7/14
49.	Martinez et al., (2016)	Y	Y	N	Y	N	N	N	Y	Y	N	Y	N/A	NA	Y	7/14
50.	Martinez et al., (2017)	Y	Y	N	Y	C/D	N	N	Y	Y	N	Y	N/A	NA	Y	7/14
51.	Mesdaghinia et al. (2021)	Y	Y	Y	Y	N	Y	Y	Y	Y	N	Y	CD	Y	Y	11/14
52.	Mousa et al., (2021)	Y	Y	Y	Y	N	N	N	Y	Y	N	Y	CD	NA	N	7/14
53.	Noviyanti et al., (2021)	Y	Y	CD	Y	N	N	N	Y	Y	N	Y	CD	NA	N	6/14
54.	Oner et al., (2018)	Y	Y	Y	Y	0	Y	Y	Y	Y	N	Y	Y	CD	Y	11/14
55.	Ortiz-Lopez et al., (2021)	Y	Y	CD	Y	Y	N	N	Y	Y	N	Y	N/A	CD	N	7/14
56.	Ozyilmaz & Taner (2018)	Y	Y	Y	Y	CD	N	N/A	Y	Y	N	Y	N/A	NA	Y	8/14
57.	Parlar-Kilic et al., (2021)	Y	Y	Y	Y	N	N	N	Y	Y	N	Y	N/A	NA	N	7/14
58.	Polat et al., (2018)	Y	Y	Y	Y	Y	N	N	Y	Y	N	Y	N/A	NA	Y	9/14

59.	Rainer & Schneider, (2020)	Y	Y	N	Y	N	N	N	Y	Y	N	Y	N/A	NA	N	6/14
60.	Raemer et al., (2015)	Y	Y	Y	Y	N	Y	Y	Y	NA	N	Y	Y	Y	Y	11/14
61.	Reyhanoglu & Akin, (2020)	Y	Y	C/D	Y	N	N	N	Y	Y	N	Y	N/A	NA	C/D	6/14
62.	Richard et al., (2021)	Y	Y	N	Y	Y	N	N	Y	Y	N	Y	CD	NA	Y	8/14
63.	Ridley et al., (2021)	Y	Y	C/D	Y	C/D	Y	Y	Y	Y	Y	Y	Y	Y	N	11/14
64.	Roussin et al. (2018)	Y	Y	Y	Y	N	N	N	Y	Y	Y	Y	Y	Y	Y	11/14
65.	Schwappach (2018)	Y	Y	N	Y	N	N	N	Y	Y	N	Y	N/A	NA	N	6/14
66.	Schwappach & Niederhauser (2019)	Y	Y	N	Y	N	N	N	Y	Y	N	Y	N/A	NA	C/D	6/14
67.	Schwappach & Richard (2018)	Y	Y	N	Y	N	N	N	Y	Y	N	Y	N/A	NA	N	6/14
68.	Schwappach & Sendlhofer (2019)	Y	Y	N	Y	N	N	N	Y	Y	N	Y	N/A	NA	N/A	6/14
69.	Schwappach et al. (2018)	Y	Y	N	Y	N	N	N	Y	Y	N	Y	Y	NA	N	6/14
70.	Seren et al., (2018)	Y	Y	Y	Y	N	N	N	Y	Y	N	Y	N/A	NA	N/A	6/14
71.	Toy et al., (2019)	Y	Y	CD	Y	N	N	N	Y	Y	N	NA	N	NA	Y	6/14
72.	Voogt et al., (2019)	Y	Y	CD	Y	N	N	N	Y	Y	N	Y	N/A	NA	N	6/14
73.	Weiss et al., (2017)	Y	Y	C/D	Y	N	Y	N	N/A	NR	N	Y	Y	NA	N	6/14

74.	Yalcin & Baykal (2019)	Y	Y	CD	Y	Y	NR	NR	Y	Y	Y	NR	Y	Y	N	9/14
75.	Zhang et al., 2021	Y	Y	Y	Y	N	N	N	Y	Y	N	Y	Y	NA	Y	9/14
76.	Zhou et al., (2021)	Y	Y	Y	Y	Y	N	N	Y	Y	N	Y	N/A	NA	CD	8/14
